# Direct Observation of Dynamic Lithium Diffusion Behavior in Nickel-Rich, LiNi_0.8_Mn_0.1_Co_0.1_O_2_ (NMC811) Cathodes Using *Operando* Muon Spectroscopy

**DOI:** 10.1021/acs.chemmater.2c03834

**Published:** 2023-05-08

**Authors:** Innes McClelland, Samuel G. Booth, Nirmalesh N. Anthonisamy, Laurence A. Middlemiss, Gabriel E. Pérez, Edmund J. Cussen, Peter J. Baker, Serena A. Cussen

**Affiliations:** † Department of Materials Science and Engineering, The University of Sheffield, Sheffield S1 3JD, United Kingdom; ‡ The Faraday Institution, Quad One, Harwell Campus, Didcot OX11 0RA, United Kingdom; § ISIS Neutron and Muon Source, Science and Technology Facilities Council, Rutherford Appleton Laboratory, Harwell Campus, Didcot OX11 0QX, United Kingdom

## Abstract

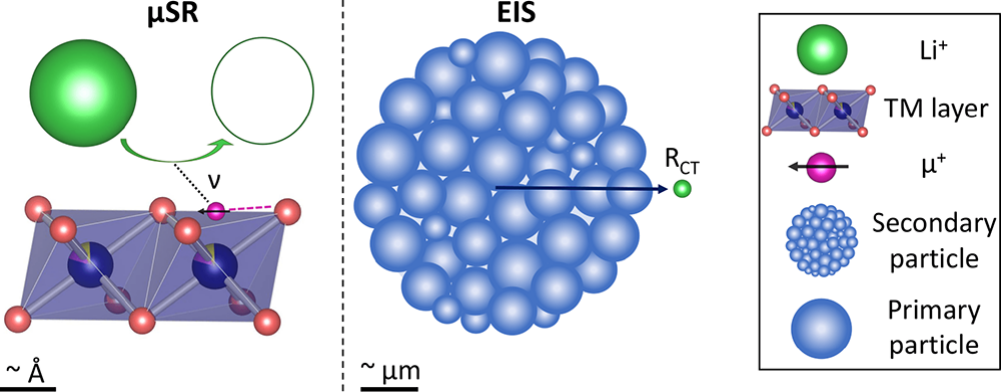

Ni-rich layered oxide cathode materials such as LiNi_0.8_Mn_0.1_Co_0.1_O_2_ (NMC811) are widely tipped as the next-generation cathodes for lithium-ion batteries. The NMC class offers high capacities but suffers an irreversible first cycle capacity loss, a result of slow Li^+^ diffusion kinetics at a low state of charge. Understanding the origin of these kinetic hindrances to Li^+^ mobility inside the cathode is vital to negate the first cycle capacity loss in future materials design. Here, we report on the development of *operando* muon spectroscopy (μSR) to probe the Å-length scale Li^+^ ion diffusion in NMC811 during its first cycle and how this can be compared to electrochemical impedance spectroscopy (EIS) and the galvanostatic intermittent titration technique (GITT). Volume-averaged muon implantation enables measurements that are largely unaffected by interface/surface effects, thus providing a specific characterization of the fundamental bulk properties to complement surface-dominated electrochemical methods. First cycle measurements show that the bulk Li^+^ mobility is less affected than the surface Li^+^ mobility at full depth of discharge, indicating that sluggish surface diffusion is the likely cause of first cycle irreversible capacity loss. Additionally, we demonstrate that trends in the nuclear field distribution width of the implanted muons during cycling correlate with those observed in differential capacity, suggesting the sensitivity of this μSR parameter to structural changes during cycling.

## Introduction

The promise of high capacity offered by the Ni-rich layered oxide cathode composition LiNi_0.8_Mn_0.1_Co_0.1_O_2_ (NMC811, practical capacity >200 mAh g^–1^)^1^ is somewhat dampened by significant and irreversible capacity loss over the first cycle. While there are numerous material degradation processes that exist for NMC811, including increased Li/Ni mixing,^2,3^ surface layer reconstruction,^4,5^ particle cracking,^6^ and decomposition reactions with the electrolyte,^7,8^ a major cause of the first cycle irreversible capacity loss has been shown to be poor Li^+^ ion mobility at a low state of charge.^9−11^ Whittingham et al. have shown that this capacity loss, present across all NMC compositions, can be reduced by 80% by simply increasing the battery operating temperature.^12^ Kinetic limitations within cathode materials are pertinent;^9,13^ sluggish Li^+^ diffusion has also been proposed as a cause of overpotential growth, resulting in reduced cathode utilization.^14,15^ Rate capability is increasingly paramount for fast charging battery applications such as electric vehicles, and such mobility problems inevitably lead to a poor rate capability of the cathode. High rate performance depends on many cell characteristics,^16^ including mass transport, percolating Li^+^ pathways, adhesion to the current collector, and solid–electrolyte interface formation, but the fundamental performance limitations rely on the lithium diffusion coefficients (*D*_Li_) within the electrode materials.^17^

It is important to consider, and distinguish, the differing Li^+^ ion mobility properties within both the bulk and surface regions in NMC811.^4,18^ A reconstructed rock-salt layer, formed at the primary particle surface through the aggressive reduction of Ni^4+^ to Ni^3+^ by the electrolyte at high voltage,^19,20^ has been found to display significantly lowered Li^+^ ion diffusion compared to the bulk,^21^ and although the surface layer is limited in thickness, this can result in a bottleneck for Li^+^ transport between primary particles. Furthermore, lattice mismatch between the bulk and the surface rock-salt at high SOC (because of the well-established lattice contraction above 4.2 V) has been reported as a primary cause of fatigue degradation in the bulk.^4^ While much interest rightly focuses on the harsh degradation processes that occur at high voltage in NMCs,^5,19^ the fundamental kinetic limitations during discharge that cause the first cycle irreversible capacity loss,^12^ still present in restricted voltage windows,^11^ are less well understood.

To uncover the limiting factors for ionic mobility, a direct characterization of the electrochemical phenomena, which occur during cycling (i.e., an *operando* measurement), is highly desirable, as some subtle effects may be hidden for *ex situ* measurements in relaxed cells.^22,23^ Although for many techniques, this is nontrivial,^24^ it is a logical next step to afford an improved understanding of battery operation under realistic conditions. Previous studies of NMC811 have employed the enhanced capabilities of *operando* techniques to study structural,^25^ diffusional,^13^ and spectroscopic^26^ properties to excellent effect. To this end, the comparison between multiple *operando* techniques, which probe slightly different Li^+^ ion mobility properties, is beneficial for completeness. Muon spectroscopy (μSR) has become a well-established local probe of diffusion properties, suitable for mobile ions including Li^+^, Na^+^, K^+^, and Mg^2+^.^27−30^ To date, most μSR studies of ionic diffusion have focused on pristine, as-prepared materials, such as for NMC811.^31^ Such studies provide useful fundamental properties although, importantly, they often measure fully lithiated cathode compounds, where ionic hopping occurs via interstitial sites or neighboring defects. While one study has recently reported *operando* μSR on a LiCoO_2_ half-cell, a comprehensive comparison with electrochemical techniques remains lacking.^32^

The ionic mobility properties during cycling depend heavily on the vacancies within available pathways, site blocking, surface layers, and layer spacing. While impedance spectroscopy (EIS) or galvanostatic intermittent titration technique (GITT) methods can provide the chemical diffusion coefficient as a function of SOC, such measurements cover the entire cell and are not direct measurements of fundamental diffusion within a material, as they also account for factors such as surface layers and the morphology/size of primary particles. The development of an *operando* approach to μSR, as reported here, enables non-invasive characterization of the variation in diffusion properties within an electrode material during operation.

Focusing on the first charge/discharge cycle, this study provides a thorough characterization of the Li^+^ diffusion properties of the cathode material LiNi_0.8_Mn_0.1_Co_0.1_O_2_ (NMC811) within a cycling battery cell. Complimentary *operando* X-ray diffraction, EIS, and GITT are used to understand the variation in structure/property relationships in NMC811 during electrochemical cycling for comparison with μSR. Our data provides generally good agreement between EIS, GITT, and μSR, with the key observation that the trends in Li^+^ diffusion rate deviate at depth of discharge, indicating that the sluggish kinetics associated with first cycle capacity loss is not a property of the bulk material.

## Experimental Methods

### Materials and Synthesis

The hydroxide precursor Ni_0.8_Mn_0.1_Co_0.1_(OH)_2_ was prepared using a co-precipitation method inside a controlled stirred tank reactor at pH 11. Stoichiometric quantities of NiSO_4_·6H_2_O (>98%, Sigma), MnSO_4_·*x*H_2_O (>98%, Sigma), and CoSO_4_·7H_2_O (>99%, Sigma) were weighed and dissolved in a solution of NH_4_OH and deoxygenated water. The reaction was completed overnight under continuous agitation and N_2_ gas flow inside the vessel, which was held at 60 °C. The mixture was allowed to settle before being washed using deoxygenated water. This process was repeated multiple times before the mixture was dried in an oven to yield the brown precursor powder. To lithiate the hydroxide precursor, Ni_0.8_Mn_0.1_Co_0.1_(OH)_2_ and LiOH·H_2_O were weighed and ground together thoroughly for 30 min in an agate mortar. A 10% weight excess of LiOH·H_2_O was used to account for losses during calcination. Calcination was performed in two steps: first 450 °C for 12 h followed by 30 min of grinding in an Ar-filled glovebox and then 850 °C for 12 h before immediate transfer to the glovebox while hot to minimize air exposure. Both steps were completed in a tube furnace under an O_2_ atmosphere with a ramp rate of 4 °C/min.

### Characterization

X-ray diffraction measurements were performed using a Rigaku Miniflex with a Cu Kα X-ray source with wavelength 1.5406 Å. *Operando* X-ray diffraction was performed using an ECC-Opto-Std test cell (EL-cell) at a rate of C/50 (based on a practical capacity of 200 mAh g^–1^) using a 5 × 10 mm Kapton window, which was transparent to X-rays. The EL-Cell was assembled using a freshly cut lithium disk covered by a glass fiber separator and then a NMC-811 composite cathode deposited on a mesh aluminum current collector. Electrolyte (LiPF_6_ in ethylene carbonate:ethyl methyl carbonate [50:50 v/v] with 2% vinylene carbonate additive, Solvionic, France) was added using a syringe. Sequential Pawley fits were employed to determine lattice parameters. SEM measurements were conducted with an FEI Inspect F50 high-resolution electron microscope using an accelerating voltage of 5 kV.

Electrochemical measurements for EIS and μSR were conducted in half cells *vs* lithium metal anodes (16 mm diameter and 0.25 mm thick, PI-KEM, UK), using a liquid electrolyte. The active material, NMC811, was mixed thoroughly in an agate mortar with conductive carbon and PTFE binder in an ABC wt % ratio of 70:20:10, respectively. *In situ* impedance measurements were collected using a Biologic VMP-300 potentiostat, while *operando* electrochemistry was conducted using a Biologic VSP potentiostat. For the *in situ* EIS experiment, a powder cathode with a 12 mg cm^–2^ active material loading was used in a Swagelok type cell and cycled at a rate of C/20. Galvanostatic intermittent titration technique (GITT) measurements were performed on NMC811|Li in a 2032 coin cell (CES) using a thin film electrode assembled with an ABC ratio of 91.5:4.5:4.0. Galvanostatic current pulses of 5 mA g^–1^ were applied for 30 min before a 2 h relaxation step. Cells were cycled between 3.0 and 4.5 V.

Muon spin relaxation measurements were conducted on the EMU spectrometer at the ISIS Neutron and Muon Source^33^ in a specifically designed BAM (Battery Analysis by Muon) cell.^34^ A description of ionic diffusion using μSR has been given previously by our group.^30^ The current collectors used were 100 μm-thick 306 L grade austenitic steel. This grade is non-magnetic and thin enough to not interfere significantly with the muon signal. Pre-cut Li discs were used as the anode material, with approximately 400 μL of electrolyte. A thick cathode was necessary to ensure adequate muon implantation in the region of interest. Therefore, each cell was prepared with an active material loading of around 70 mg cm^–2^ (area of ∼2 cm^2^), with additional separators and electrolyte used to ensure appropriate wetting. Continuous μSR measurements were taken to 20 million positron detection events in the order of 0, 10, and 20 Gauss longitudinally applied fields during (de)lithiation. These three field measurements were grouped to form one distinct measurement in this experiment, allowing the collection of 71 points over the first cycle. A rate of C/20 was used (current density of 10 mA g^–1^), and two pauses were necessary due to muon beam outages. All measurements were conducted at 300 K. For *operando* measurements, the electric potential applied to the cell is much smaller than the local fields the muon will experience at its stopping site in the NMC811 structure, meaning the applied electric field will not induce muon motion. Mantid was used for all μSR data analysis.^35^

## Results and Discussion

### NMC811 Synthesis and Characterization

**Figure 1:**
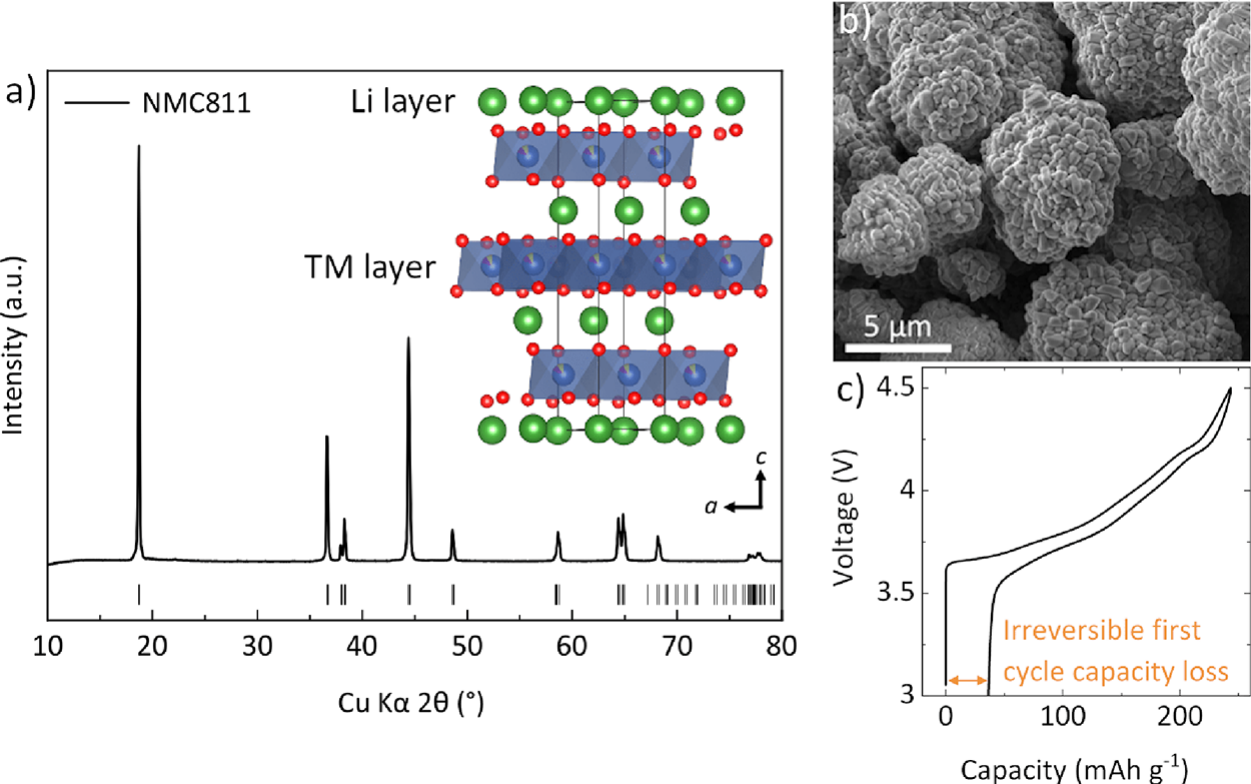
(a) X-ray diffraction pattern of synthesized LiNi_0.8_Mn_0.1_Co_0.1_O_2_ (NMC811) for which a Rietveld refinement can be found in Figure S1. The layered Li and transition metal (TM) structure is shown, with the unit cell outlined. (b) Scanning electron micrograph showing spherical secondary particles. (c) First cycle of a NMC811|Li half-cell at C/20 between 3.0 and 4.5 V, showing the irreversible first cycle capacity loss.

A pH-controlled stirred tank reaction was used to produce a hydroxide precursor, which was then thoroughly mixed with LiOH·H_2_O and calcined under oxygen flow to yield the cathode material, LiNi_0.8_Mn_0.1_Co_0.1_O_2_ (NMC811). An X-ray powder diffraction pattern of the resultant NMC811 is shown in Figure 1a, confirming a single phase *R*3̅*m* structure with a (003)/(104) peak ratio of ∼1.7, indicating low cation mixing.^36^ Rietveld refinement (Figure S1) was performed to validate the quality of the sample and determine lattice parameters of *a* = *b* = 2.87376 (8) and *c* = 14.2099 (3) Å. A scanning electron micrograph is displayed in Figure 1b, which depicts the NMC811 material with spherical secondary particles between 5 and 10 μm consisting of primary particles several hundred nanometers in diameter. This is typical of polycrystalline NMC811 synthesized via the co-precipitation method. Figure 1c provides the first galvanostatic charge/discharge cycle for polycrystalline NMC811 at a C/20 rate, with a first charge capacity of 243 mAh g^–1^ and a first discharge capacity of 207 mAh g^–1^, illustrating an irreversible first cycle capacity loss of 14.8%. A loss of capacity within the first charge/discharge cycle has been observed for all NMC-type compositions.^12^ To understand how the Li^+^ diffusion properties within different parts of the material during the first cycle may influence this irreversible capacity loss, an *operando* approach to μSR was developed and compared to electrochemical characterization techniques EIS and GITT.

### *Operando* μSR – Experimental Details

To understand the local properties Li^+^ diffusion within the bulk during cycling, we have developed an *operando* method to measure muon spectroscopy (μSR) as a function of state of charge. These measurements provide a local measurement of the site–site Li^+^ hopping rate by utilizing implanted, spin-polarized, muons.^29,30^ μSR measurements are therefore representative of the ionic mobility in the bulk, independent of electrode–electrolyte surface area, and less sensitive to cathode–electrolyte interface formation or surface reconstructions. Usefully, the technique allows the isolation of Li^+^ transport in the active material from that within the electrolyte since diffusion rates in liquid electrolytes (∼10^–6^ to 10^–7^ cm^2^ s^–1^)^37^ are outside the motional range probed by the muon lifetime (∼10^–8^ to 10^–13^ cm^2^ s^–1^).^30^

**Figure 2:**
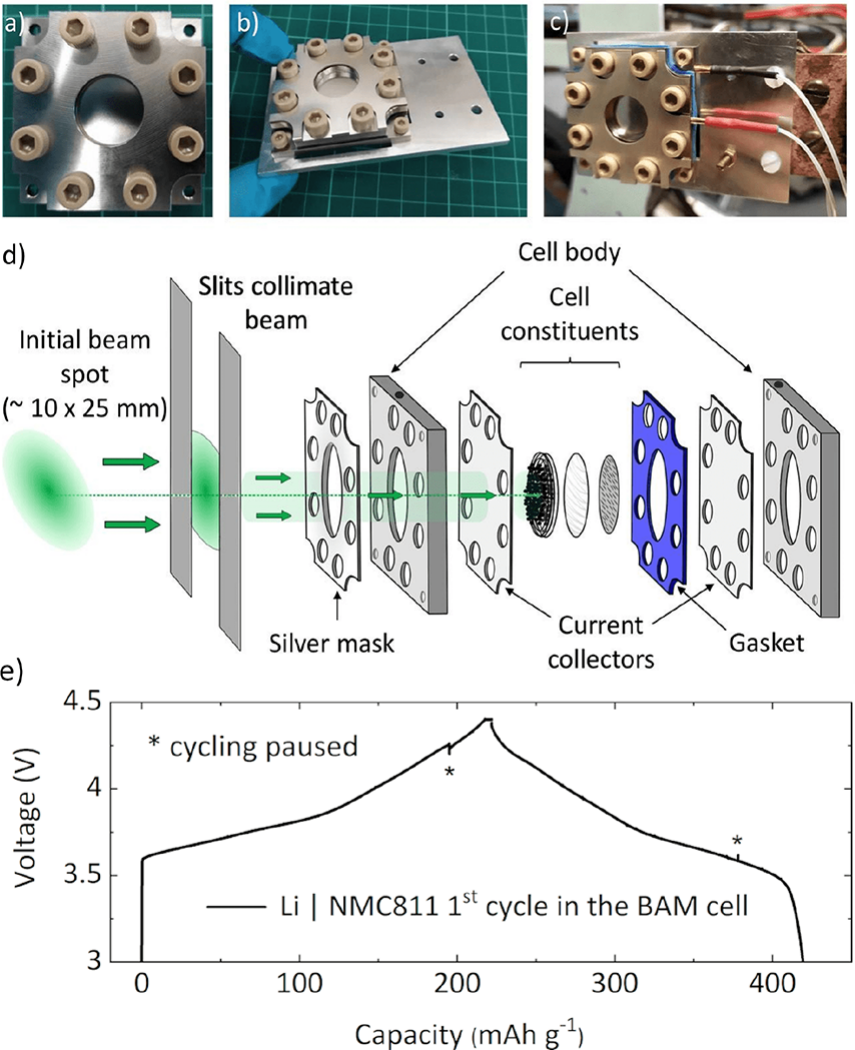
(a, b) Pictures showing the BAM (Battery Analysis by Muon) cell. (c) Cell fixed on the EMU spectrometer at the ISIS Neutron and Muon Source. (d) Experimental schematic showing the beam penetration into the cathode. (e) Charge/discharge cycle of a Li|NMC811 configuration in the BAM cell, recorded during the *operando* μSR experiment.

The experiment was enabled by the development of a custom electrochemical cell. Figure 2a–c pictures the assembled BAM (Battery Analysis by Muon) cell, which possesses a stainless steel inspection window of diameter 18 mm to allow an ample beam penetration area. The cell components are expanded to show the experimental set up in Figure 2d. Figure 2e displays the first cycle of the Li|NMC811 cell on the beamline. The BAM cell is found to display very similar electrochemical performance to other cell types, although we note that the high mass loading required for the μSR experiment (∼70 mg cm^–2^) induces a small overpotential due to an unavoidable increase in internal resistance over more commonly used mass loadings (Figure S2). Further details on cell assembly are given in the Supporting Information.

Utilizing the *operando* cell, we achieved a first charge capacity of 222 mAh g^–1^ and first discharge capacity of 198 mAh g^–1^ (Figure 2e). The muon stopping profile in the cathode was determined from the thickness and density of the components; it was also simulated using the stopping range of ions in matter (SRIM) program,^38^ with the result shown in Figure S3. The range curve on the EMU beamline implants muons between 100 and 200 mg cm^–2^ as an areal density. The 100 μm-thick steel current collector provides an areal density of around 80 mg cm^–2^. Therefore, the muon ensemble will pass through the current collector and implant into the mixture of cathode (∼100 mg cm^–2^) and electrolyte (∼165 mg cm^–2^). Although a large cathode mass was used, muons implant into everything they encounter within the cell. Consequently, the relaxing signal coming from the cathode active material, *A*_KT_, accounts for ∼1/6th of the measured initial amplitude, *A*_0_ (eq 1). Li^+^ transport via the electrolyte is too fast to be detectable within the muon lifetime^39^ and Li^+^ surface sorption through the carbon additive is far too low in volume fraction to significantly influence the signal, meaning any contribution from this to the muon spin relaxation is minimal. An exponential relaxation, *P*_exp_, was added to the fitting function to account for muon stopping sites inside non-active battery components (i.e., carbon/binder). All muons will likely be stopped before reaching the separator, agreeing with the SRIM simulation. Therefore, *A*_KT_ can be confidently assumed to arise from Li^+^ transport within the NMC811 crystal structure. Any inhomogeneities across the thick electrode are averaged out in the obtained signal.

Continuous zero and longitudinal field measurements were collected to follow the Li^+^ dynamics during the first charge/discharge cycle. The data were fit in the time domain (*t*) 0.1–25 μs using a composite function containing a flat background, a dynamic Kubo–Toyabe function, and an exponential relaxation described as
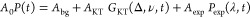
1

The component amplitudes (*A*) and the relaxation rate (λ) were held as global parameters (given in Table S2), which were fixed to the average amplitude value across all runs. The field fluctuation rate (ν) and the static field distribution width (Δ) were allowed to fluctuate. *G*_KT_ is the dynamic Kubo–Toyabe function, which in the static, zero-field limit is of the form
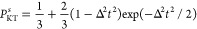
2

This can be related to *G*_KT_ as
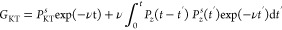
3where *t* – *t*′ is the time between collisions in the strong collision model. CCCV (constant current, constant voltage) cycling was applied to compensate for the kinetic limitations expected when using a high mass loading^40^ and to provide sufficient time for a measurement at 4.4 V.

### *Operando* μSR – Li^+^ Mobility

**Figure 3:**
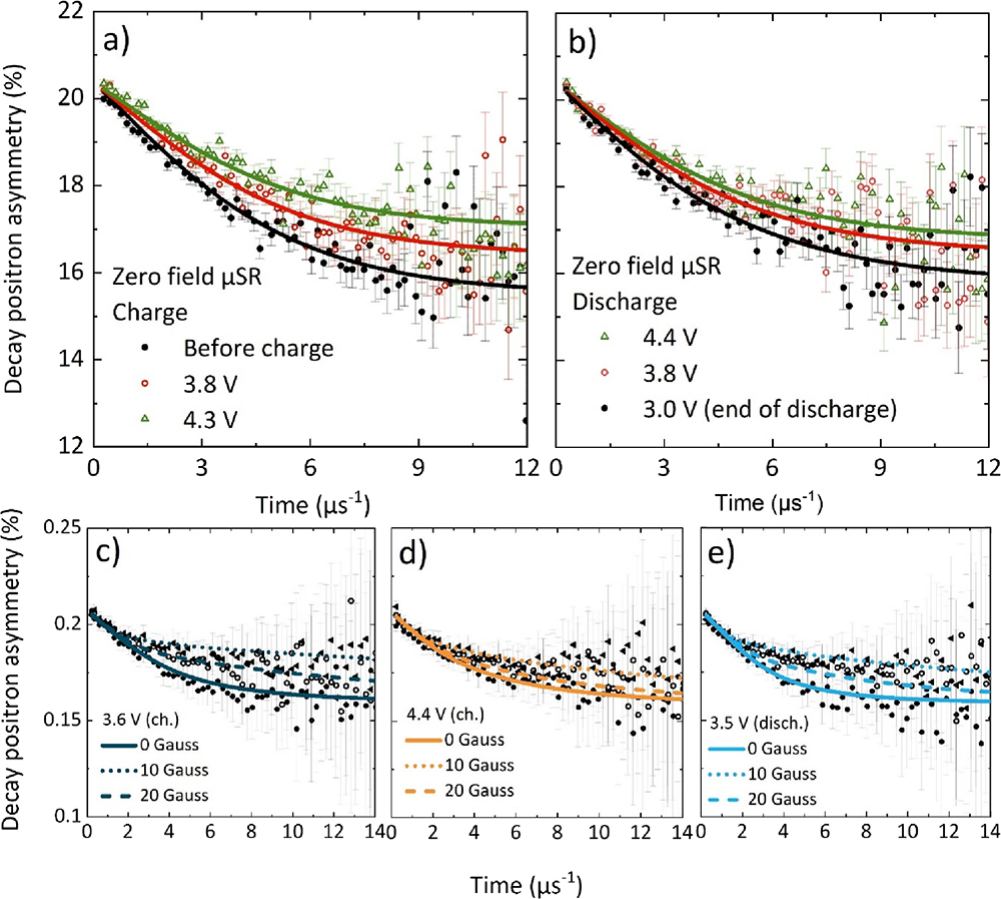
(a, b) Zero-field μSR spectra in the time range 0–12 μs at different voltages of the Li|NMC811 *operando* cell. (c) Fit curves for 0, 10, and 20 G longitudinal fields at the beginning (3.6 V, navy), (d) the top (4.4 V, orange), and (e) the end (3.5 V, light blue) of the first charge/discharge cycle. Data and fits for all voltage points can be found in Figure S4.

To illustrate how the muon signal changes during the first cycle, three zero-field measurements at selected voltages in the time range 0–12 μs are given for both the charge and discharge in Figure 3a,b, respectively. In general, the form of the relaxation will vary depending on the ratio ν/Δ;^41^Figure 3 therefore indicates faster Li^+^ ion diffusion at high SOC. Although there is a high background fraction that changes slightly with SOC, there is a noticeable decrease in relaxation of the zero-field data at the top of charge, before it increases again, to a lesser extent, during discharge. To compare all three applied longitudinal fields (0, 10, and 20 G), which are grouped together during fitting, plots are given at the beginning of the first charge (3.6 V), the top of the first charge (4.4 V), and the end of the first discharge (3.5 V) in Figure 3c–e, respectively. The form of the asymmetry does not return to its initial state after discharge (at 3.5 V), with the deviation clearer at longer timescales (≥6 μs). Fitted μSR plots at 0.1 V intervals across the first cycle are presented in Figure S4. A comparison of the zero-field fit curves, given in Figure S5, indicates that the rate of Li^+^ diffusion tends to increase over the first charge until high SOC, where it slows down. The reverse trend occurs over the course of the discharge cycle, with a large change evident below 3.7 V, indicating poor Li^+^ ion dynamics in this lower voltage region.

**Figure 4:**
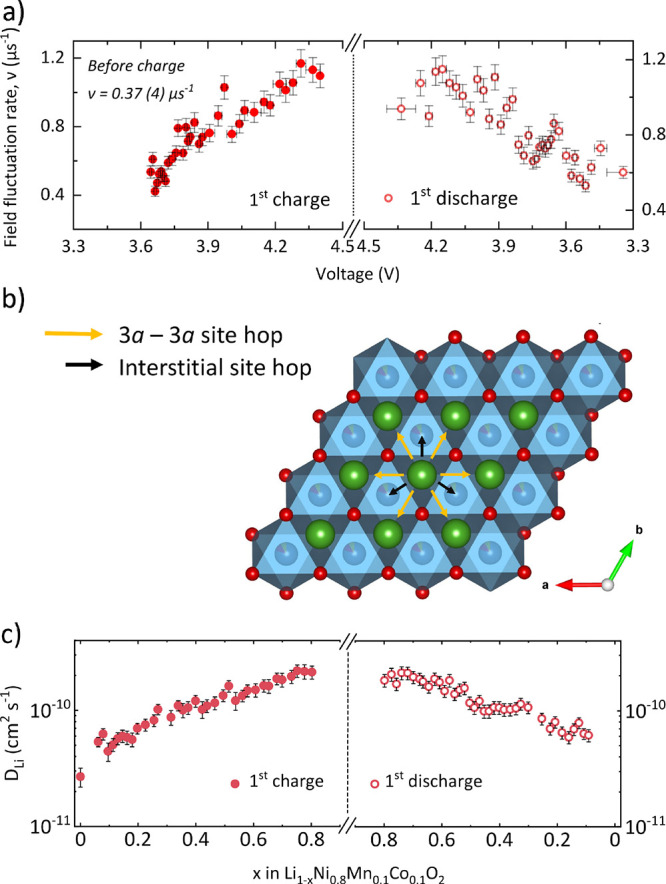
(a) Field fluctuation rate, ν, which is analogous to the ionic hopping (Li^+^ diffusion) rate in NMC811, during the first charge/discharge of a NMC811|Li cell. (b) Projection looking down the *c* axis to visualize the two possible Li hops: direct site and interstitial hopping. (c) Li^+^ diffusion coefficient, *D*_Li_, as a function of Li content in NMC811 from μSR. This can be obtained using ν and the hopping pathways depicted in panel (b).

Fitting eq 1 to the data collected during cycling tracks the evolution of two important parameters: the field fluctuation rate (ν) and the static field distribution width (Δ). As a Li^+^ ion diffuses past an implanted muon, its nuclear moment causes a change in local magnetic field, which acts to flip the muon spin. As such, the field fluctuation rate is analogous to the ionic hopping (Li^+^ diffusion) rate;^29^ to analyze the variation in Li^+^ diffusion during cycling, ν was plotted against voltage (Figure 4a). The diffusion rate coefficient, *D*_Li_, was determined using eq 4:
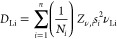
4

*N_i_* is the number of possible Li sites for diffusion in the *i*th path, *Z* is defined as the vacancy fraction (i.e., 1 – Li occupancy) for the *i*th site, *s_i_* is the site-site hopping distance, and ν is the field fluctuation rate obtained *via* μSR data fitting. Values of *Z* were found using a Coulomb counting method, assuming minimal side reactions during cycling, and the site–site hopping distances were found from the lattice parameters deduced from *operando* X-ray diffraction, shown in Figures S6 and S7. In NMC811, there are two available pathways for Li^+^ diffusion: directly from 3*a* to 3*a* sites, or through interstitial sites (Figure 4b). At full Li site occupancy (i.e., 0% SOC), the 3*a* to 3*a* pathway is unavailable as there are no vacant sites available for diffusion, and hence the interstitial pathway is dominant. As charging begins and Li^+^ is extracted from the cathode structure, 3*a* vacancies appear and 3*a*-to-3*a* site–site hopping becomes increasingly influential. At OCV, with close to full Li occupancy in the cathode, *D*_Li_ is at its lowest due to the difficulty of Li^+^ ion diffusion to a vacant site (Figure 4c). The value of *D*_Li_ found before cycling of ∼3.4 × 10^–11^ cm^2^ s^–1^ compares well to that found by a previous μSR study of NMC811 (reported as 2.9 × 10^–11^ cm^2^ s^–1^).^31^

**Figure 5:**
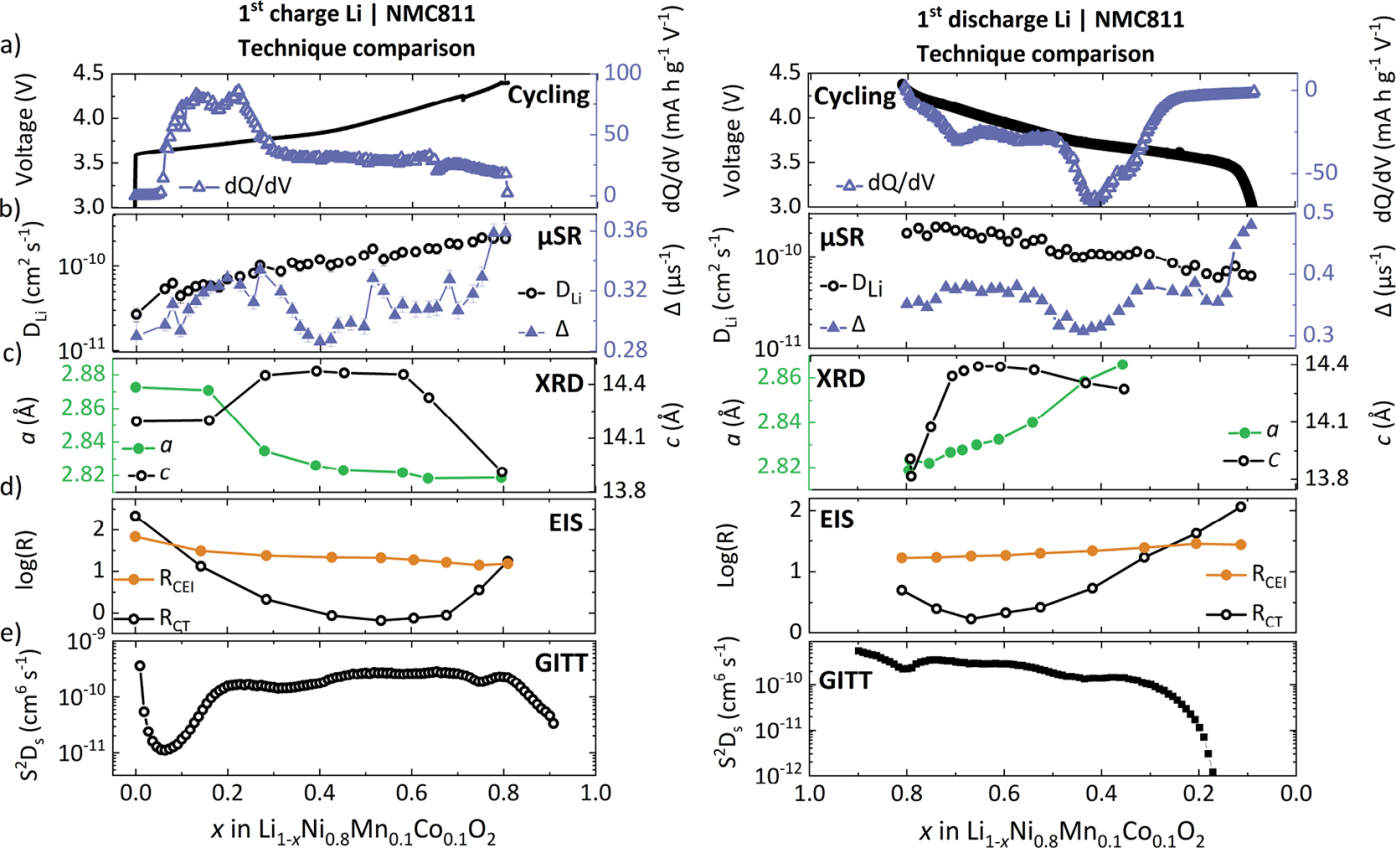
Dataset comparison for the first charge and discharge cycle of various Li|NMC811 half cells displaying (a) the first cycle of the BAM cell during the *operando* μSR experiment, alongside the differential capacity plot, (b) the change in the self-diffusion coefficient, *D*_Li_, and the static field distribution width, Δ. Further data and experiment details for each technique can be found in the Supporting Information. (c) *Operando* X-ray diffraction experiment showing the change in lattice parameters during charging and discharging, (d) the change in charge transfer resistance (*R*_CT_) and cathode electrolyte interface resistance (*R*_CEI_) measured by electrochemical impedance spectroscopy, and (e) the change in surface-independent chemical diffusion coefficient for Li^+^ measured using the galvanostatic intermittent titration technique.

*D*_Li_ is observed to rise rapidly to above 5 × 10^–11^ cm^2^ s^–1^ at the beginning of the first charge as the voltage rises to ∼3.6 V and starts to plateau. The beginning of charge sees the fastest rate of increase, agreeing with NMR,^26^ GITT,^42^ and EIS^43^ reported in the literature. As the battery continues to charge with further Li^+^ ions leaving the cathode, *D*_Li_ increases steadily to around 10^–10^ cm^2^ s^–1^ at *x* = 0.4 (*x* in Li_1–*x*_Ni_0.8_Mn_0.1_Co_0.1_O_2_). The change is due to the gradual increase in the Li-layer spacing, which acts to decrease the activation energy for ionic hopping,^42,44^ and the increasing vacancy fraction on the 3*a* site. *D*_Li_ continues to climb steadily at a slower pace until around *x* = 0.75, where it reaches a maximum and begins to fall, correlating with the high voltage event seen in the d*Q*/d*V* plot at 4.2 V. This is a consequence of the collapse in the Li-layer spacing, causing the reduction in unit cell volume as observed by X-ray diffraction, shown in Figure 5c. This tightening of diffusion pathways during the unit cell contraction will counteract the activation energy decrease for Li^+^ diffusion from the increasing vacancy fraction. Upon discharge, shown in Figure 4c, the trend is largely reversed: the hopping rate and consequently *D*_Li_ increase slightly at the beginning of discharge as the *c* axis rapidly expands again, upon initial re-intercalation of Li^+^, reaching a maximum at ∼*x* = 0.75. As Li^+^ is re-inserted, the vacancy fraction decreases and acts to reduce the available pathways for diffusion. *D*_Li_ then reduces steadily throughout the discharge, reaching a value slightly higher than that found at the beginning of charge. This measured trend for NMC811 appears to be in line with the trend seen in another *operando* μSR study on LiCoO_2_,^32^ with both studies showing reversibility in *D*_Li_. However, without parallel electrochemical performance between the studies, it is difficult to draw detailed comparisons.

Interestingly, although the reduction in Li^+^ mobility toward depth of discharge has been reported for NMC compositions,^9−12^ this is not apparent in the μSR data. This discrepancy motivates the usage of further characterization techniques to determine the root cause of the sluggish kinetics at depth of discharger comparison.

### EIS Measurements

After evaluating the dynamic lithium diffusion behavior of the material at a local level, *in situ* impedance spectroscopy (EIS) and galvanostatic intermittent titration technique (GITT) were employed as probes of bulk diffusion across the full cathode material. This approach was designed to address the observed differences in Li^+^ diffusion dynamics reported for layered materials using different techniques.^42,44^ Matching our *operando* μSR methodology, for EIS and GITT, a NMC811|Li half-cell was prepared and cycled at a rate of C/20. EIS measurements were taken at regular intervals (Figure 5d, Figure S7). We note that when comparing cells between characterization techniques, there is expected to be small differences in the voltage point at which structural and electron transfer phenomena occur during cycling due to the inherent differences between the cycling programs and cell configuration required for each method (as seen in Figure S2). The collected spectra were fit using an equivalent circuit consisting of two [*RQ*] elements (a resistor, *R*, and constant phase element, *Q*, in parallel) in series with another resistor and a Warburg element. This model is very similar to that previously used for NMC cells^42−45^ and described the data well (Figure S9). The first resistor represents the solution resistance, while the first *RQ* element, visible as the first semi-circle in Figure S8b, describes the cathode–electrolyte interface (CEI) effects present in the cell. The third component is assigned to the resistance of charge transfer through the NMC particles themselves (*R*_CT_). Figure S8b shows the change in the Nyquist plot over Li^+^ (de)intercalation, while Figure 5d displays the trend in the fit values of the two resistance components.

*R*_CEI_ is found to initially drop as charging begins, likely due to the removal of adventitious lithium carbonate impurities,^43,44^ before reducing steadily during charge. The reverse is true upon discharge, although it does not climb back to its initial value. This may be caused by an increase of the electrode–electrolyte surface area as a result of cracking in the secondary particles at high potential, allowing electrolyte infiltration to increase the contact area.^6^*R*_CT_ reaches a minimum around *x* = 0.5 where the second semicircle at lower frequency disappears, which agrees well with other electrochemical analyses of NMC811 by GITT and EIS.^42,46^ Above *x* = 0.7, *R*_CT_ rises rapidly, and the second semicircle reappears in the Nyquist plot: this effect is well correlated with the contraction of the *c* axis measured by *operando* X-ray diffraction (Figure 5c).^18,44^ Indeed, interfacial mismatch between the bulk and surface rock-salt phases as a result of lattice expansion and contraction during cycling has been described as a primary cause of fatigue degradation in the bulk,^4,18,25^ which evidently increases *R*_CT_. At the end of the first cycle, *R*_CT_ returns to a high value, which is less than at *x* = 0 (before charge).

### GITT and Technique Comparison

The trend in Li^+^ mobility was also evaluated via the galvanostatic intermittent titration technique (GITT). Figure S10a displays the cycling profile for the GITT experiment, which involved a 30 min current pulse followed by a 2 h relaxation period (inset). Since an accurate surface area for the porous electrode is difficult to measure, the surface independent diffusion coefficient (S^2^D_s_) was determined and is displayed in Figure 5e. The methodology for determining S^2^D_s_ is given in the Supporting Information. Since the charge transfer resistance estimated by EIS is inversely proportional to the diffusion coefficient, the trend in *R*_CT_ with SOC appears to correlate well with S^2^D_s_ found via GITT and with the *D*_Li_ obtained through *operando* μSR measurements. In the GITT experiment, the first points collected tend to be an overestimate since the cell does not fully relax before the next current pulse is applied.^44^ The trends in S^2^D_s_ and *R*_CT_ therefore agree as the mobility of Li^+^ ions improve as charging begins and site vacancies are formed. A minimum in *R*_CT_ and a maximum in S^2^D_s_ are reached during charging at ∼*x* = 0.5, where Li^+^ mobility is at its highest due to the availability of appropriate pathways and wide interlayer spacings. This trend in Li^+^ diffusion during charge largely compares well with that determined by μSR and EIS, with the slowest rates at low SOC and an increasing Li^+^ mobility as the charging progresses. There is a small decrease observed for S^2^D_s_ at *x* between 0.7 and 0.8, which correlates with the high voltage event in the d*Q*/d*V* profile seen in Figure 5a.^44^ All three techniques (μSR, EIS, and GITT) display a decrease in Li^+^ mobility above *x* = 0.7, where surface layer formation, in combination with the lattice contraction (observed by X-ray diffraction in Figure 5c), acts to restrict transport properties. Above *x* = 0.7, the Li^+^ mobility determined by μSR displays a smaller reduction than that observed by electrochemical techniques (Figure 5b,d,e). This may indicate that surface layer formation, or interfacial mismatch, which has a much higher influence over the electrochemical methods, will affect the overall diffusion rate to a greater extent than the lattice contraction does for the bulk.

The EIS data given in Figure 5d demonstrate that *R*_CT_ does not return to its initial value after one cycle. This apparent reduction in *R*_CT_ at the end of the discharge cycle has been observed and explained by Janek and co-workers^6^ as a consequence of an increase in electrode–electrolyte surface area via the infiltration of liquid electrolyte into cracks in secondary particles, which can occur at high potentials. The electrode surface area is assumed to be constant for the electrochemical determination of the Li^+^ ion diffusion coefficient; thus, the measured values during discharge may be considered to be an overestimate. Curiously, the discrepancy between diffusion rates at depth of discharge observed by EIS is reversed for GITT, which displays a large drop in Li^+^ mobility below *x* = 0.3. This trend has been observed in other studies,^42^ and while it is poorly understood, it is given as a major cause of the first cycle irreversible capacity loss for NMC compositions.^9−12^ At low SOC during discharge, S^2^D_s_ is around an order of magnitude lower than the minimum during the charging step, while for EIS, *R*_CT_ is lower at the end of discharge than at the beginning of the first charge. The inherent assumption made here is that both electrochemical techniques (GITT, EIS) are expected to have a similar dependence on the cathode surface area; this assumption requires further attention.

μSR is found to measure very similar values of Li^+^ mobility during the charge and discharge, showing no evidence for hysteresis (Figure S11). Interestingly, the reversibility seen for μSR is not reflected by GITT results at depth of discharge, indicating differences in diffusion properties across parts of the material. In particular, bulk Li^+^ diffusion, measured by μSR, remains at a higher level at low SOC on discharge, comparable to the data collected during charge. The independence of μSR measurements to the active material surface area allows us to confidently assign the trends in diffusion rate seen primarily to the bulk. However, in the GITT data, below *x* = 0.25 on discharge the diffusion rate is found to drop rapidly. When analyzing the properties of both techniques, these results indicate that surface Li^+^ diffusion effects are dominating the trend seen by GITT. The comparison of such techniques, both for fundamental and functional material properties, is thus important to understand the regions of ionic mobility limitations during cycling.

The significant (∼2 orders of magnitude) drop in *D*_Li_ in the discharged state observed by GITT is not reflected in the μSR data, indicating that it is not a fundamental property of the bulk material but is instead related to processes occurring at the cathode particle surface. It is important to note that the chemical diffusion coefficient from electrochemical techniques is related to the diffusion coefficient from μSR via a thermodynamic factor, which is not constant over cycling.^47^ Nonetheless, the differences between the trends in diffusion are inherent to the technique used; muon implantation is volume averaged, meaning that limiting factors for overall ionic migration such as large resistances at particle surfaces^5^ are not influential. The μSR data here describes the diffusion coefficient from a local level (i.e., site–site hopping) within the bulk NMC811 particles, while electrochemical techniques probe longer-range transport properties. The difference in results between characterization techniques therefore suggests that there is a difference in diffusion properties between the particle bulk and surface during the first cycle. Significantly hindered Li^+^ diffusion is not observed in the particle bulk (by μSR) at full depth of discharge, indicating that surface effects are dominating the overall trend seen by GITT. This highlights the importance of combining both surface and bulk sensitive techniques for effective characterization of these underpinning properties.

### *Operando* μSR – Muon Environment

**Figure 6:**
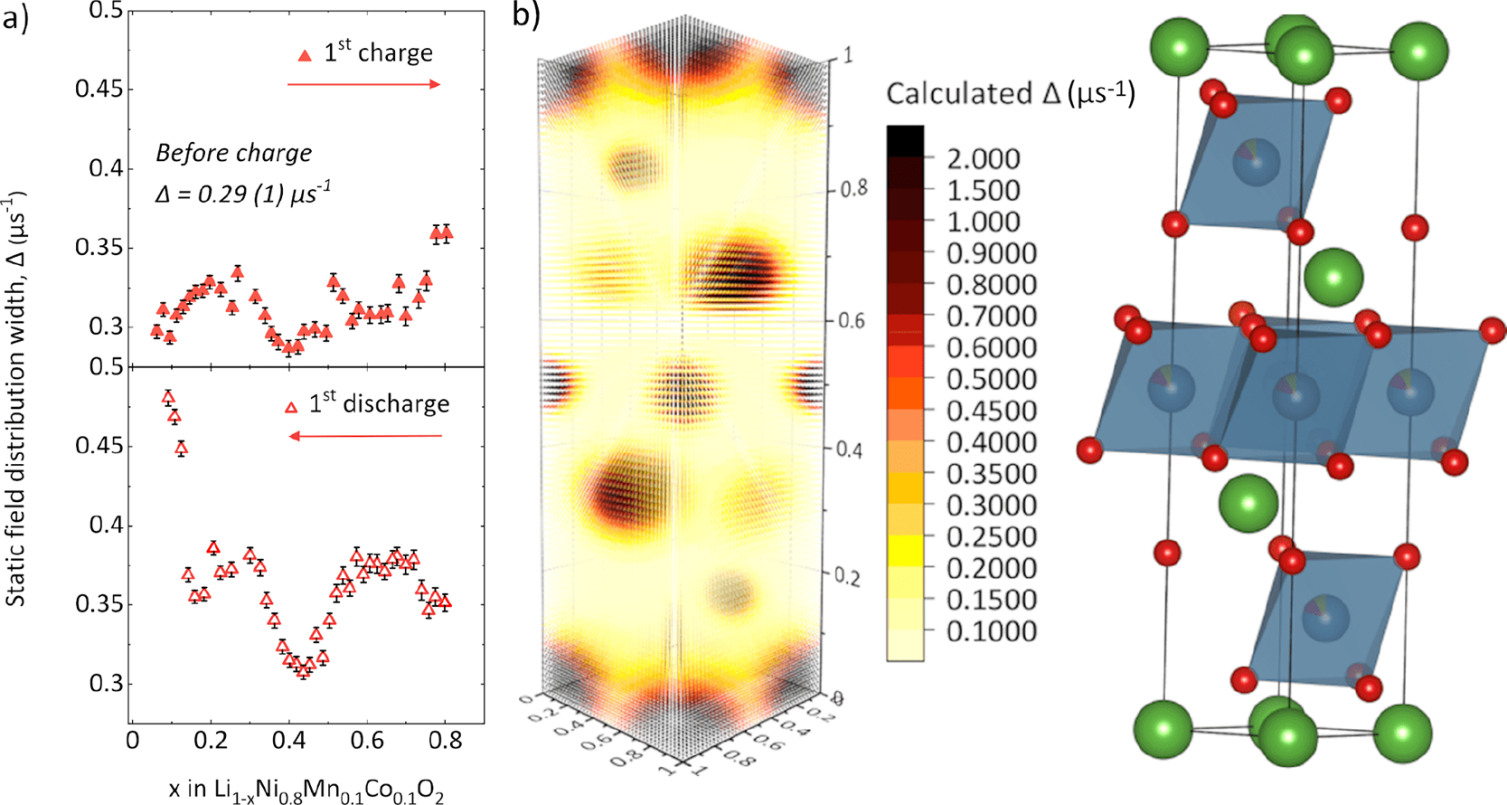
Static field distribution width, Δ, of the muon as a function of lithiation state of NMC811 during the first cycle. (b) Map of Δ values in the unit cell at full Li occupancy as predicted by dipolar field calculations. Visually evident is the greater influence of Li on Δ than the transition metals. The muon stopping site is likely in the transition metal layer.^48^ The black regions at atomic sites correspond to Δ values ≫ 2 μs^–1^.

In addition to the field fluctuation rate (ν), the static field distribution width (Δ) was obtained via μSR and represents the proximity and strength of nuclear magnetic moments near the muon stopping site (nuclear moments and abundances of the relevant isotopes for NMC811 are given in Table S5.) Figure 6a displays graphically the variation in Δ during the first charge/discharge process. Muons prefer to reside near the electronegative oxygen ions, forming a weak O−μ bond of length ∼1 Å,^30^ and Δ provides a sense of the local environment near this site. For NMC811, the effect of Ni and O on Δ is negligible. In contrast, Li, Co, and Mn have a much greater influence on Δ, although Co and Mn have a lower occupancy (see Table S5). Given this, Δ would be expected to decline over the first charge as Li^+^ leaves the cathode structure. Curiously, this effect is not seen experimentally, and Δ appears dependent on the lithiation state. Such dependence may indicate that the change in TM–O bond length during battery operation alters the average muon position within the structure, causing muon to experience an altered distribution of nuclear magnetic moments. To validate these results, the stopping site of the muon must be understood (see Figure 6b and Figure S12).

To narrow down the possible muon sites, dipolar field calculations (eq S2) were performed at varying SOC and compared to experimental values, which is discussed in the Supporting Information. While many muon sites are plausible at full Li occupancy, to match with the experimentally determined Δ, our calculations strongly suggest that the muon prefers to reside in the transition metal (TM) layer. This explains the lack of reduction in Δ during the charge process (Figure 6a); although it may be expected that the reduced Li occupancy would lower the field distribution width experienced by the muon, the preferred muon site indicates a stronger sensitivity to changes within the TM layer. Indeed, Forslund *et al*. used DFT calculations to determine the muon site in NaNiO_2_ as being within the NiO_2_ plane and subsequently extended the applicability of this result to other layered oxide materials.^48^

The static field distribution, Δ, is seen to resemble a clear trend with the lithiation state of the cathode, indicating a sensitivity of this parameter to the structural changes occurring within the material. Curiously, the features in Δ are reminiscent of peaks in the d*Q*/d*V* plot, with a broad feature observed during charging at ∼30% SOC, and a sharp increase in Δ above 70% SOC, which aligns with the high voltage event also seen in d*Q*/d*V* (Figure S12). While Δ is dependent on various parameters such as Li content, lattice parameters, and the muon stopping site, this correlation between the trend in d*Q*/d*V* and Δ warrants further future investigation to assign features in the experimental Δ values to specific structural events. The work we present here is a starting point for *operando* muon investigations to be applied in conjunction with complementary experimental and computational techniques to explore how the muon may be used as a structural probe in operating electrochemical systems.

## Conclusions

*Operando* μSR measurements of ionic diffusion correlate structural and diffusional changes in the NMC811 cathode during cycling, made possible through the design of a custom-built cell now available for the community to use. The Li^+^ diffusion coefficient rate in bulk NMC811 increases rapidly between 0% and 30% SOC, before slowing to a steady increase during delithiation and finally dropping off after 75% SOC. This reduction in diffusion at higher states of charge correlates with the collapse of the Li interlayer spacing as observed by XRD. On discharge, this trend in diffusion rate is reversed as Li site occupancy increases. At depth of discharge, the Li^+^ diffusion rate does not decrease fully to the level observed for the pristine NMC811. We observe that for electrochemical probes of Li^+^ diffusion, a variation at depth of discharge, with EIS R_CT_ values lower than for the initial pristine state and GITT measurements, shows a significant drop off in *D*_Li_. By comparison, μSR is independent of long range and surface effects within the cell, providing a short-range, volume-averaged measurement of the site–site hopping within the bulk material. The elevated *D*_Li_ values at depth of discharge, relating to the reduced Li site occupancy *vs* the pristine material, reveal that sluggish Li^+^ diffusion is not inherent to the bulk material but more likely linked to changes at the surface of the cathode during the first cycle. As such, doping and coating processes, focusing on stabilizing the surface of the cathode material, are likely to be most effective at retaining the capacity of the cathode. We demonstrate here that *operando* μSR is an excellent complement to electrochemical and spectroscopic data to provide a more comprehensive assessment of transport properties in energy storage materials.

## Supporting Information Available

Rietveld refinement results, impedance fitting example and parameters, *operando* X-ray diffraction data, electrochemical comparison between cell types, simulated muon stopping profile, GITT methodology, raw μSR data figures and fitting parameter table, μSR fitting parameter comparison with d*Q*/d*V*, dipolar field calculations and muon site discussion (PDF)
