# Supplementary material for: Direct Observation of Dynamic Lithium Diffusion Behavior in Nickel-Rich, LiNi0.8Mn0.1Co0.1O2 (NMC811) Cathodes Using Operando Muon Spectroscopy
Source: Chem Mater. 2023 May 8;35(11):4149–58. doi: 10.1021/acs.chemmater.2c03834 (PMC10268956; doi:10.1021/acs.chemmater.2c03834)
Supplement: Supplementary file 1 — cm2c03834_si_001.pdf [file cm2c03834_si_001.pdf]

Supporting Information

Direct Observation of Dynamic Lithium Diffusion Behaviour in Nickel-Rich, $LiNi_{0.8}Mn_{0.1}Co_{0.1}O_2$ (NMC811) Cathodes using O*perando* Muon Spectroscopy

Innes McClelland,[1,2,3] Samuel G. Booth,[1,2] Nirmalesh N. Anthonisamy,[1,2] Laurence A. Middlemiss,[1,2] Gabriel E. Pérez,[2,3] Edmund J. Cussen,[1,2] Peter J. Baker,[2,3] and Serena A. Cussen*[1,2]

[1] Department of Materials Science and Engineering, The University of Sheffield, Sheffield, S1 3JD, United Kingdom

[2] The Faraday Institution, Quad One, Harwell Campus, OX11 0RA, United Kingdom

[3] ISIS Neutron and Muon Source, Science and Technology Facilities Council, Rutherford Appleton Laboratory, Harwell Campus, Didcot, OX11 0QX, United Kingdom

Rietveld refinement of as-synthesized NMC811

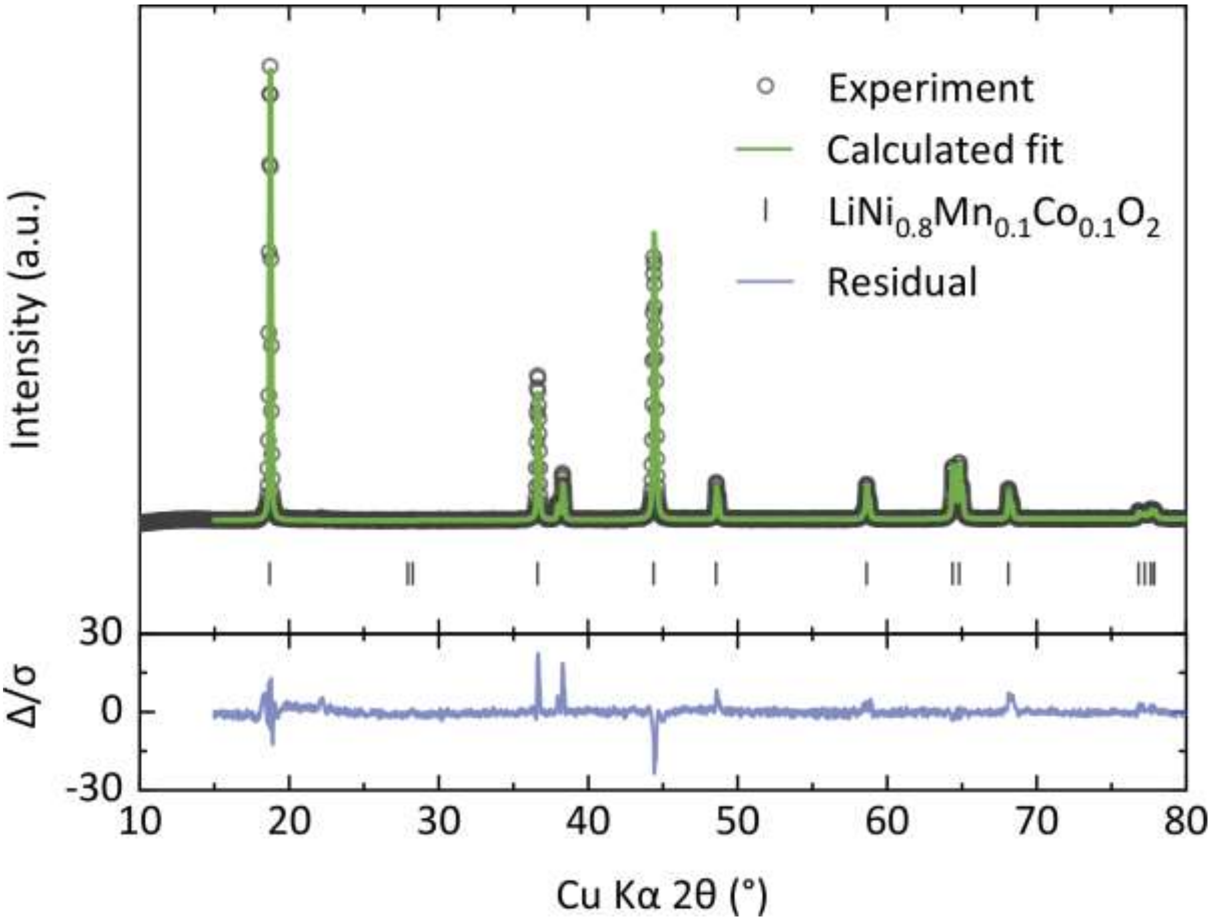

Figure S1. Rietveld refinement of as-synthesized $LiNi_{0.8}Mn_{0.1}Co_{0.1}O_2$ to a $R\bar{3}m$ layered oxide crystal structure after calcination at 850 ˚C for 12 h in an O_2 atmosphere. A weighted residual of 1.81% was found.

Table S1. Various structural parameters obtained by a Rietveld refinement of the as-synthesized NMC811 sample.

**Rietveld Refinement (from Figure S1)**	
Material	$LiNi_{0.8}Mn_{0.1}Co_{0.1}O_2$
Space Group	$R\bar{3}m$
a, *b*	2.87376 (8) Å
c	14.2099 (3) Å
α, β, γ	90˚, 90˚, 120˚
Volume	101.630 (5) Å^3
R_{WP}	1.81%

BAM (Battery Analysis by Muon) *operando* cell assembly

To achieve a reasonable signal during the *operando* experiment, a significant mass of active material (≥100 mg) was required to ensure adequate muon implantation. The carbon content of the prepared cathode powders (70:20:10, NMC811:C:PTFE wt.%) was high to ensure sufficient electronic conduction across the large area/mass. The devised methodology for this experiment involved the usage of ~50 mg of cathode powder in layers, packed down using a spatula. Small glass-microfibre separator semicircles were used alongside separator rings to hold additional electrolyte within the cell to fully wet all cathode particles and enable high-rate cycling, although large contact areas between cathode layers were retained. This cell assembly procedure was found to display comparable performances to standard low mass loading coin/Swagelok type cell configurations over the first few cycles (i.e., ~200 mAh g^{-1} first discharge capacity) and produced consistent cycling results.

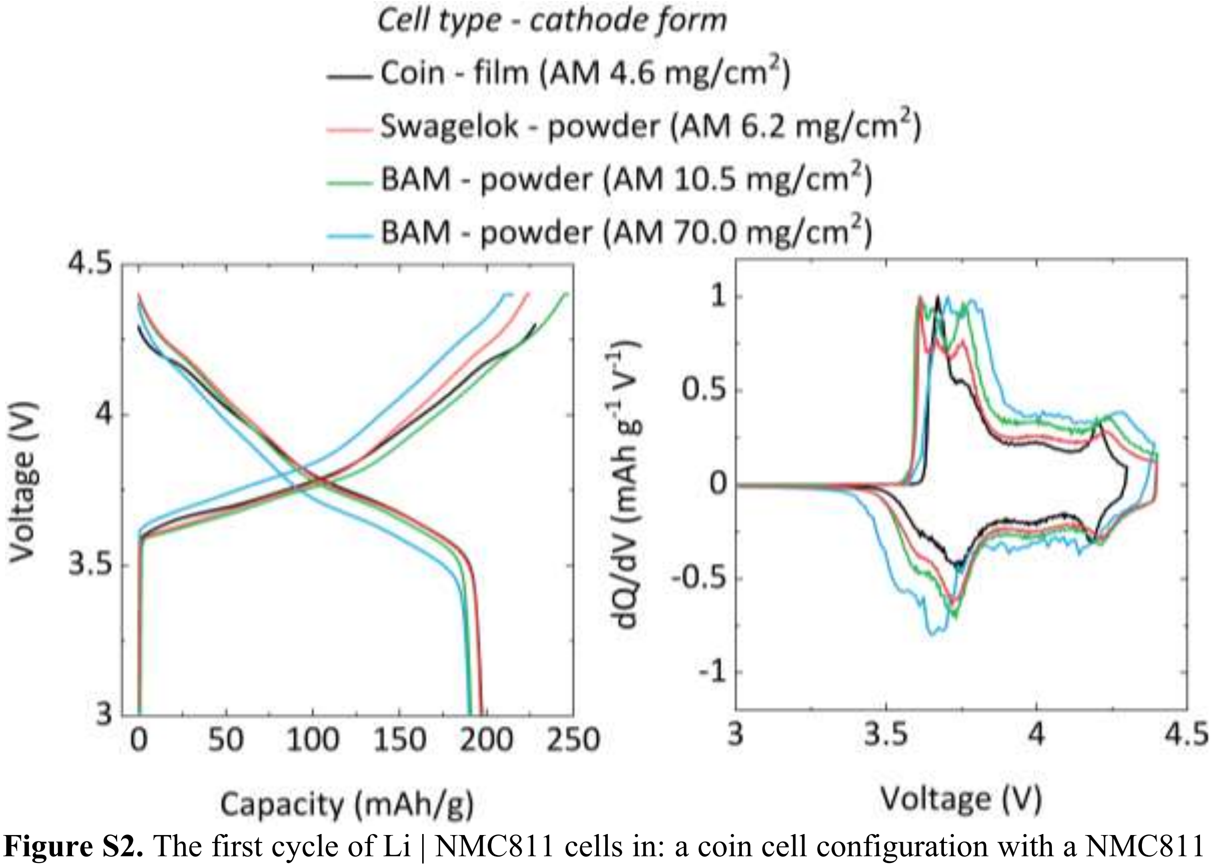

Figure S2. The first cycle of Li | NMC811 cells in: a coin cell configuration with a NMC811 cathode film (active material [AM] loading 4.6 mg/cm^{-2}), a Swagelok configuration with an active material (AM) loading of 6.2 mg cm^{-2}, a BAM configuration with an AM loading of 10.5 mg cm^{-2}, and a BAM configuration with an AM loading of 70.0 mg cm^{-2}. Differential capacity (dQ/dV) plots are also shown for each cell. Note that the coin cell was cycled only to 4.3 V for this cell.

Figure S2 displays a comparison of different cell types and cathode forms. As can be clearly seen, the first cycle is very comparable between the coin, Swagelok, and BAM cells, highlighting the reproducibility of electrochemistry in our developed *operando* cell. Normalised dQ/dV plots also show very similar peak positions. As the active mass loading is increased to a level required for *operando* μSR measurements (~70 mg cm^{-2}) there is a small overpotential noticeable in the cycling and dQ/dV profile. This is expected given the unavoidable increase of internal resistance as a consequence of the greater mass loading. As such, caution is expelled when comparing datasets from different measurement techniques on different cells, as the voltage of structural and electron transfer events during cycling will be slightly shifted.

Muon Spin Relaxation Measurements

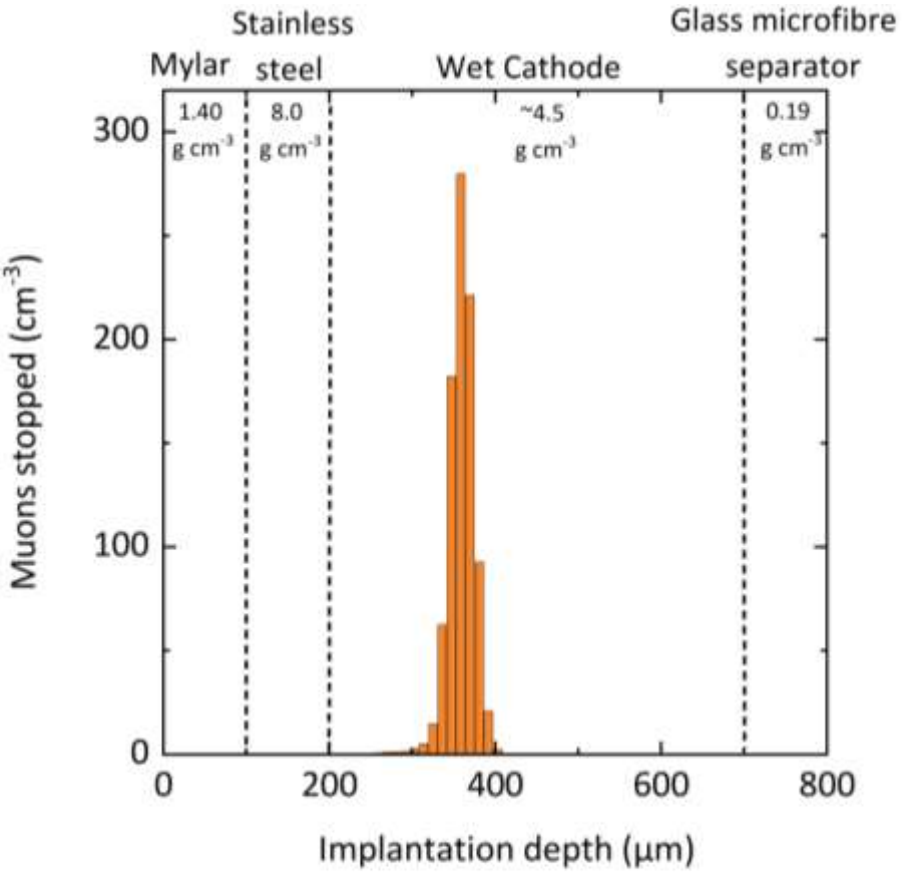

Figure S3. Stopping profile of muons in the BAM cell for the *operando* experiment, as simulated by the SRIM program. The muon is approximated as a light proton for this simulation. The path of the muon in the cell can be visualised in Figure 2d, where the stainless steel current collector is directly before the cathode. For this simulation, the cathode is assumed to be homogeneously interspersed with the electrolyte. Although the exact thickness of the cathode is not known because the cell is under compression, the mass loading is correct, which is the important factor when considering the stopping behaviour of the muons. The simulation shows that all muons are predicted to stop within the cathode layer, implanting within all its constituents. Very few muons are likely to reach the separator. We can thus be confident that most muons will stop in the cathode/electrolyte layer within the cell.

Operando μSR Fitting

Muon measurements were fitted using Eq. 1. The exponential term was added to account for the other materials contained within the *operando* cell (carbon, binder, *etc*.), which some muons will undoubtedly stop within. Due to the low volume fraction of active material, the flat background was found to be a large value of 0.159 (2). A relatively low Kubo-Toyabe amplitude of 0.034 (2) was obtained (these are displayed in Table S3), which is around 17% of the total measured amplitude. The fitting function was constrained to three separate runs, grouped as consecutive 0, 10, 20 G measurements, to form a single 'point'. All unfixed parameters were fitted globally at each point. The voltage change during the μSR measurement period was averaged out across the time duration of the measurement. This model provided nicely fitted data across the cycle, with χ^2 values largely below 1.10. The quality of the fits are good across the first cycle (Table S4) but seem to slightly deviate at extreme potentials (i.e. high and low voltage). This is indicative of the structural changes within the sample and/or large voltage range during the measurements taken across these points. Such changes cannot realistically be modelled without a detailed *ex situ* experiment on isolated samples with specific compositions: something that will be worthwhile in future work. Thus, the model used in this work, with fixed component amplitudes across the cycle, represents the most reliable method to fit this *operando* data.

Table S2. Fitting parameters for Eq. 1 to fit the *operando* muon data of an Li/NMC811 half-cell. The average amplitudes of the three components (A_{bg}, A_{KT}, and A_{exp}) were found across the full dataset and then fixed globally across the final fits. The relaxation rate λ was found for each point and fixed to an average value. Δ and ν were allowed to fluctuate freely for each point. Figures 5a and 7a show the trends in ν and Δ over the first cycle, respectively.

**μSR fits using Eq. 1**							
**Voltage**	A_{bg}	A_{KT}	**Δ (μs⁻¹)**	**ν (μs⁻¹)**	A_{exp}	**λ (μs⁻¹)**	χ^2
1st charge							
OCV	0.159 (2)	0.034 (2)	0.29 (1)	0.37 (4)	0.0130 (1)	0.713 (2)	1.104
3.644 (5)			0.297 (4)	0.53 (4)			0.986
3.654 (5)			0.311 (4)	0.61 (4)			0.999
3.664 (5)			0.294 (4)	0.42 (3)			0.974
3.674 (5)			0.308 (4)	0.47 (3)			1.033

3.682 (5)			0.313 (4)	0.52 (3)			0.986
3.692 (5)			0.319 (4)	0.54 (3)			0.998
3.701 (5)			0.323 (4)	0.51 (3)			1.007
3.711 (6)			0.323 (4)	0.48 (3)			1.001
3.722 (6)			0.329 (4)	0.59 (4)			0.995
3.74 (1)			0.324 (4)	0.61 (4)			0.987
3.757 (5)			0.312 (4)	0.65 (4)			0.988
3.767 (5)			0.334 (5)	0.79 (5)			0.973
3.789 (6)			0.319 (4)	0.64 (4)			0.970
3.802 (5)			0.307 (5)	0.79 (5)			0.988
3.811 (5)			0.296 (5)	0.71 (5)			0.986
3.822 (6)			0.291 (5)	0.74 (5)			0.999
3.84 (1)			0.286 (5)	0.82 (6)			0.997
3.860 (8)			0.288 (5)	0.70 (5)			1.004
3.878 (9)			0.297 (5)	0.74 (5)			0.988
3.91 (2)			0.299 (5)	0.76 (5)			0.999
3.95 (2)			0.296 (5)	0.86 (6)			0.950
3.97 (1)			0.328 (5)	1.03 (7)			0.980
4.01 (2)			0.320 (5)	0.76 (5)			0.980
4.04 (1)			0.304 (5)	0.82 (5)			0.968
4.06 (1)			0.311 (5)	0.89 (6)			1.009
4.11 (3)			0.308 (5)	0.884145			0.951
4.15 (1)			0.308 (5)	0.94 (6)			1.020
4.18 (1)			0.309 (5)	0.92 (6)			0.992
4.22 (3)			0.328 (6)	1.05 (7)			0.977
4.25 (2)			0.307 (6)	1.01 (7)			1.003
4.28 (1)			0.318 (6)	1.06 (7)			0.963
4.32 (2)			0.329(6)	1.17 (8)			0.979
4.37 (3)			0.359 (6)	1.13 (7)			0.998
4.399			0.359 (6)	1.10 (7)			0.993
1st discharge							
4.33 (6)	0.159 (2)	0.034 (2)	0.351 (5)	0.94 (6)	0.0130 (1)	0.713 (2)	0.993
4.25 (2)			0.355 (6)	1.07 (7)			1.019
4.21 (2)			0.347 (5)	0.90 (6)			1.025
4.18 (1)			0.359 (6)	1.14 (7)			1.044

4.15 (2)			0.379 (6)	1.15 (7)			1.013
4.12 (1)			0.375 (6)	1.07 (7)			1.074
4.09 (1)			0.381 (6)	1.05 (6)			1.127
4.06 (1)			0.379 (6)	1.01 (6)			1.075
4.03 (2)			0.371 (5)	0.92 (5)			1.111
3.99 (1)			0.376 (6)	1.10 (7)			1.068
3.97 (1)			0.376 (6)	1.04 (6)			1.078
3.94 (1)			0.369 (5)	0.88 (5)			1.086
3.91 (1)			0.380 (6)	1.11 (7)			1.100
3.89 (1)			0.361 (5)	0.85 (5)			1.012
3.86 (1)			0.369 (5)	0.94 (6)			1.069
3.84 (1)			0.358 (5)	0.99 (6)			1.043
3.81 (1)			0.340 (5)	0.75 (5)			1.057
3.79 (1)			0.317 (5)	0.69 (4)			1.098
3.77 (1)			0.331 (5)	0.80 (5)			1.016
3.748 (9)			0.312 (5)	0.66 (4)			1.066
3.732 (7)			0.308 (5)	0.67 (5)			1.027
3.718 (7)			0.312 (5)	0.74 (5)			1.022
3.705 (7)			0.315 (5)	0.75 (5)			1.040
3.693 (6)			0.324 (5)	0.73 (5)			1.029
3.679 (8)			0.340 (5)	0.75 (5)			1.051
3.666 (6)			0.353 (5)	0.78 (5)			1.061
3.654 (6)			0.374 (5)	0.86 (5)			1.0193
3.63 (2)			0.381402	0.82 (5)			1.045
3.60 (2)			0.373 (5)	0.69 (4)			1.081
3.576 (9)			0.370 (5)	0.58 (3)			1.093
3.560 (8)			0.386 (5)	0.68 (4)			1.084
3.54 (1)			0.357 (5)	0.57 (3)			1.045
3.51 (1)			0.355 (5)	0.53 (3)			1.034
3.49 (1)			0.369 (5)	0.63 (4)			1.079
3.45 (3)			0.448 (5)	0.73 (4)			1.149
3.34 (7)			0.469 (5)	0.60 (3)			1.118
3.1 (1)			0.481 (5)	0.59 (4)			1.156

Fitted asymmetry μSR data across the first cycle of NMC811

Charge

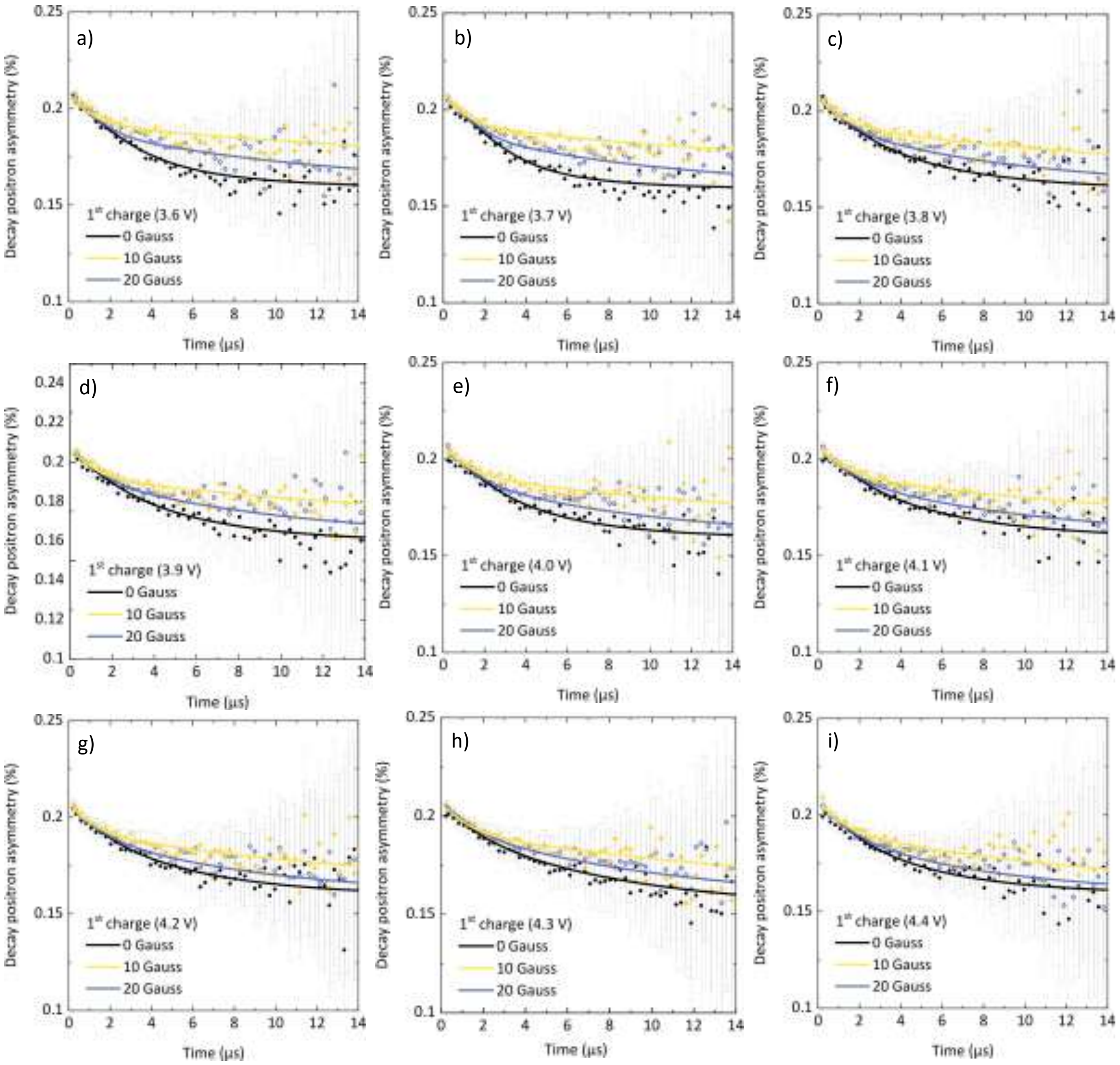

Discharge

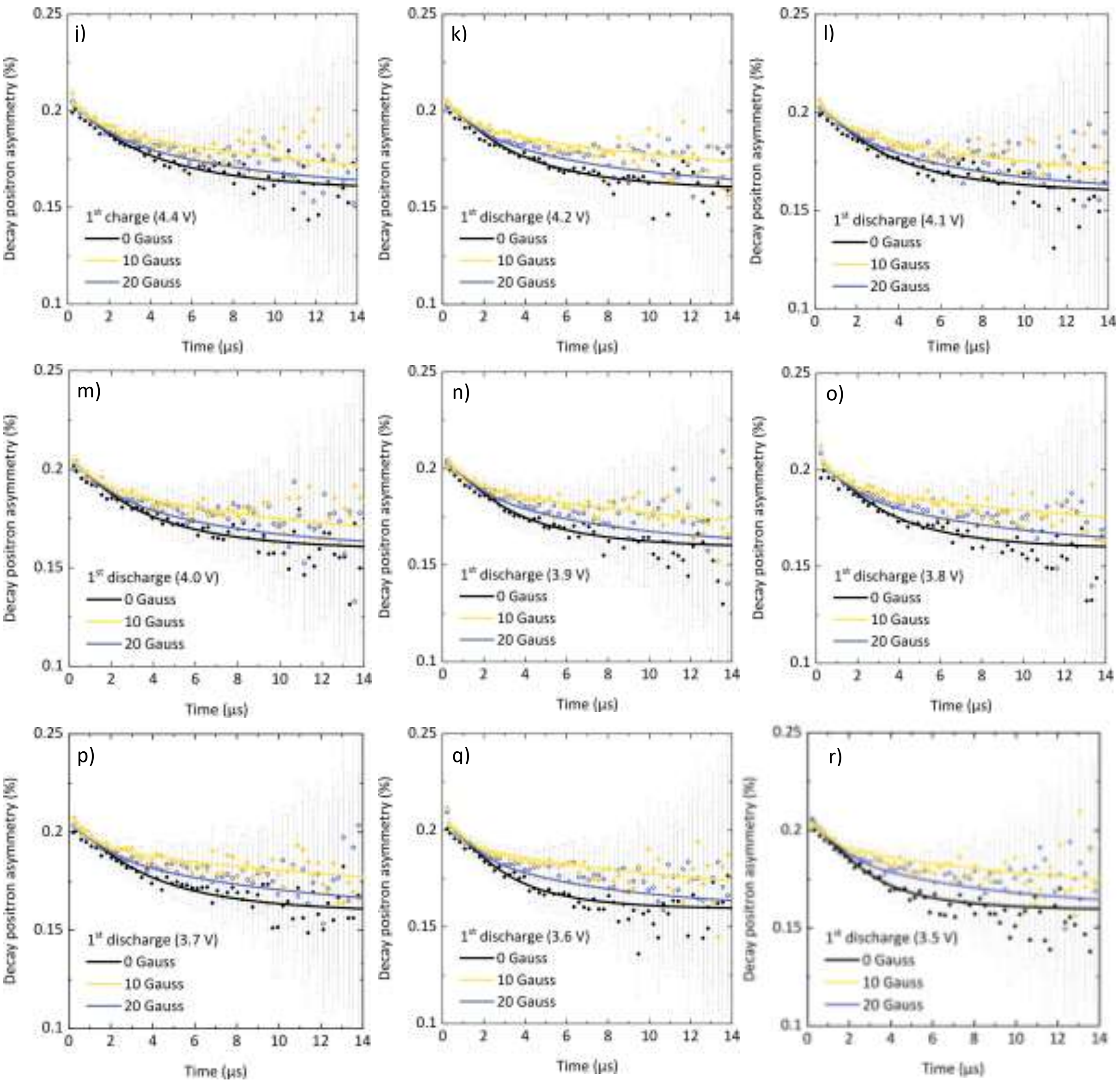

Figure S4. Fitted 0, 5, and 10 Gauss applied field strengths for a Li | NMC811 cell at 0.1 V intervals for (a-g) the first charge and (j-r) the first discharge. At all voltages a high background is seen, caused by the large amount of material in the cathode which is not NMC811, such as carbon, PTFE, and liquid electrolyte, within which muons will stop. Given the detectable timeframe for ionic diffusion seen by muons (10^{-5} - 10^{-8} s), ionic motion in the electrolyte is too fast to be detectable by µSR. From the start of the charge plateau (3.6 V) to the top (4.4 V) there is a clear increase in muon spin relaxation which suggests motional narrowing, indicative of a faster dynamical environment. This effect is largely reversed upon discharge to 3.5 V.

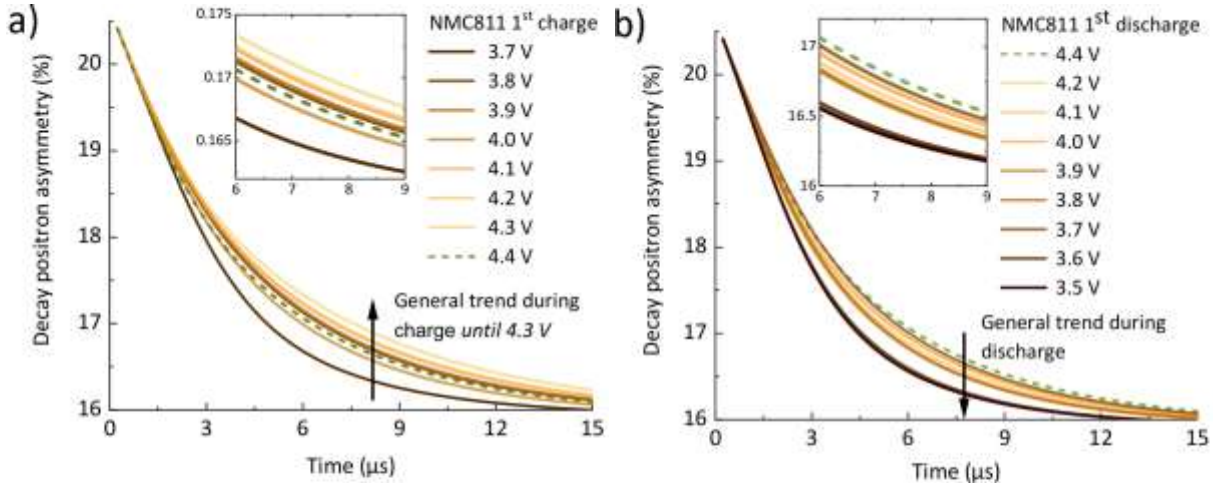

Figure S5. (a), (b) *Operando* µSR data comparing zero field (0 G) fit curves across the first charge/discharge cycle of a Li | NMC811 cell. During charging, the signal relaxation in general decreases gradually towards 4.3 V before increasing between 4.3 and 4.4 V. The reverse trend occurs over the course of the discharge cycle, with a large change evident below 3.7 V, indicating poor Li^+ ion dynamics in this lower voltage region. In fact, during both charge and discharge Li^+ mobility appears markedly slower at low SOC.

Operando **XRD experiment**

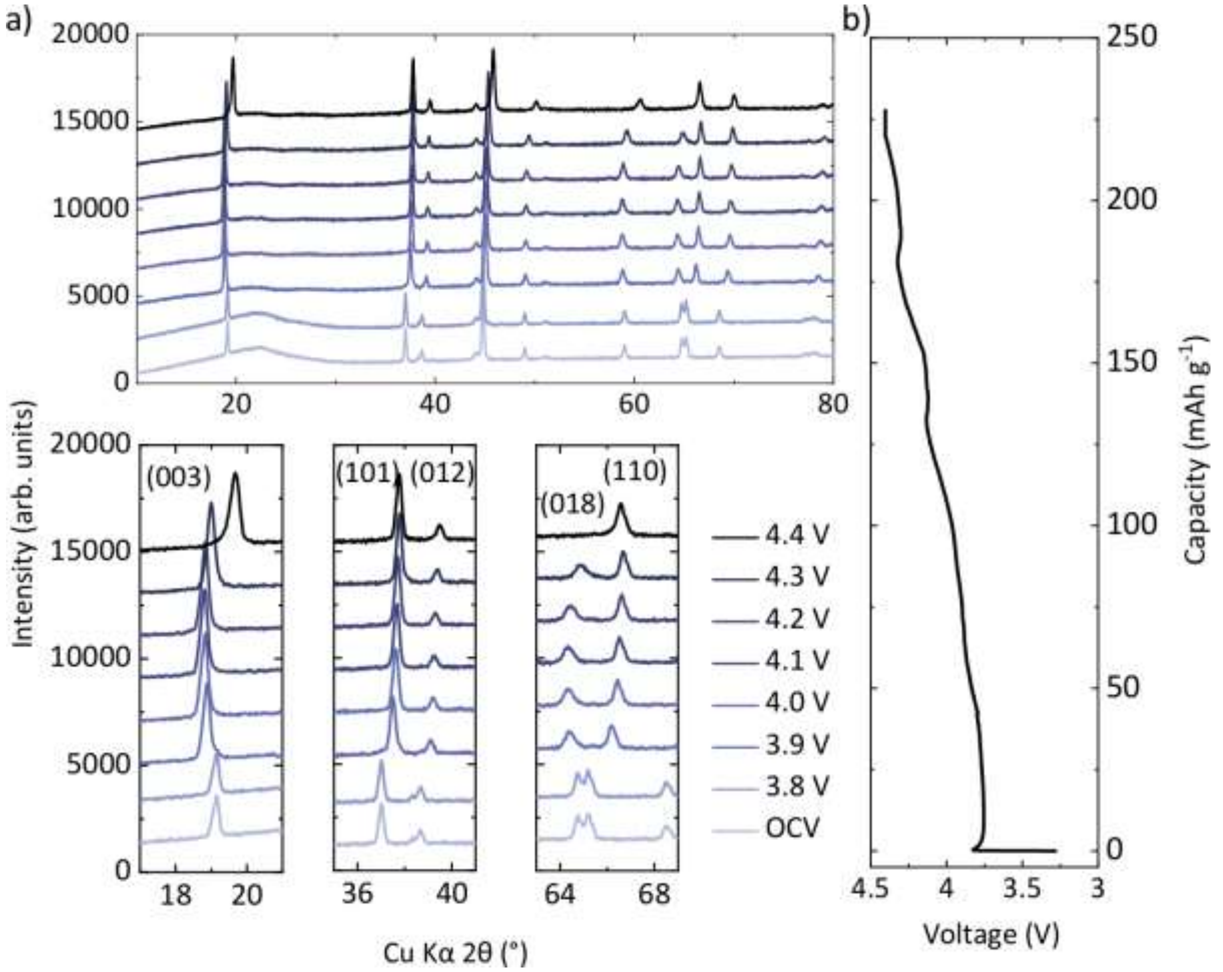

Figure S6. (a) An *operando* X-ray diffraction experiment performed using an EL-cell on a Li/NMC811 cell. The XRD pattern before charging is shown at open circuit voltage (OCV). Initially upon charging the (003) peak shifts to lower angles up to around 4.2 V, where it moves rapidly to higher angles. This suggests a contraction of the *c*-axis. The (101) peaks gradually shift to higher angles during charging, indicating a steady contraction of the *a* axis. No splitting of the (101) peak is observed indicating no distinct two-phase transition occurs. Meanwhile, the (018) and (110) peaks clearly split around 3.9 V noting a material phase transformation. (b) The first charge of the *operando* XRD cell from the results shown in (a). An initial overpotential is seen, likely due to electrode surface impurities from potential air exposure and is not predicted to significantly affect any obtained XRD patterns. The EL-cell was cycled at C/50 and achieved a charge capacity of 227 mAh g^{-1}.

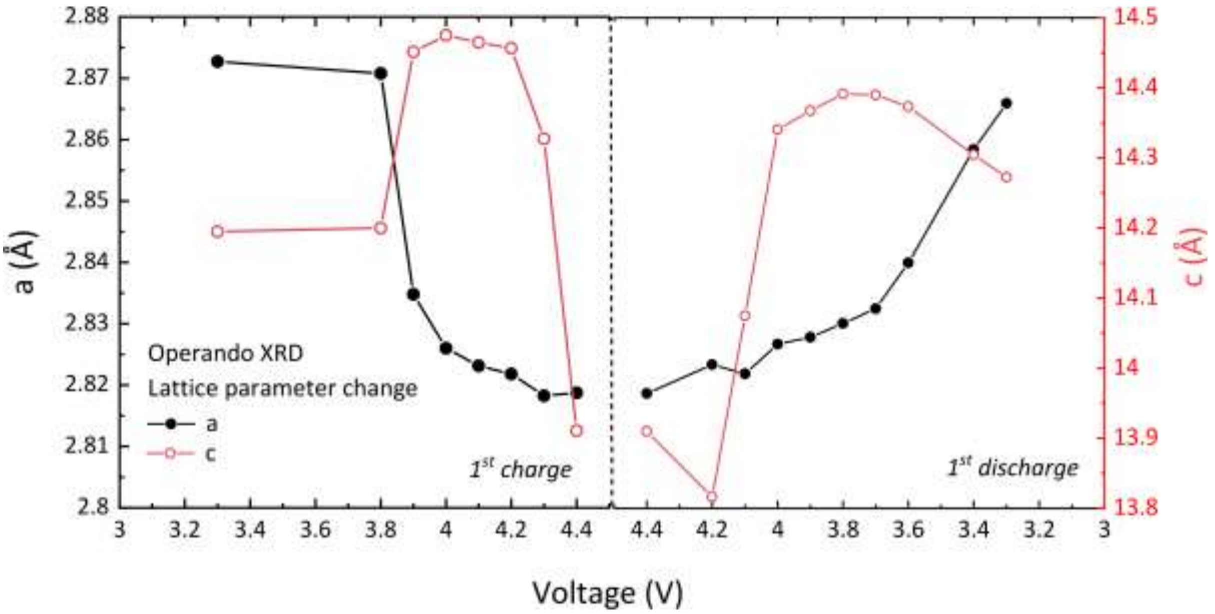

Figure S7. Lattice parameters obtained through an *operando* X-ray diffraction experiment performed using an EL-cell on a Li/NMC811 cell. Sequential Pawley fits were performed to refine the structure at each voltage point. The *c*-axis length is observed to initially expand before contracting rapidly above 4.2 V, similar to other reports.[1,2] Meanwhile, the *a*- and *b*-axes undergo a much smaller contraction during charging, followed by a steady expansion during discharging.

Table S3. Table displaying the lattice parameter trends obtained by *operando* XRD as shown in Figures S6 and S7.

**Voltage (V)**	***a*, *b* (Å)**	***c* (Å)**
	1st charge	
3.3	2.873	14.194
3.8	2.871	14.199
3.9	2.835	14.450
4.0	2.826	14.474
4.1	2.823	14.464
4.2	2.822	14.455
4.3	2.818	14.327
4.4	2.819	13.910
	1st discharge	
4.4	2.819	13.910
4.2	2.823	13.816
4.1	2.822	14.075
4.0	2.827	14.341
3.9	2.828	14.367
3.8	2.830	14.391
3.7	2.833	14.390
3.6	2.840	14.373

***In situ* EIS experiment**

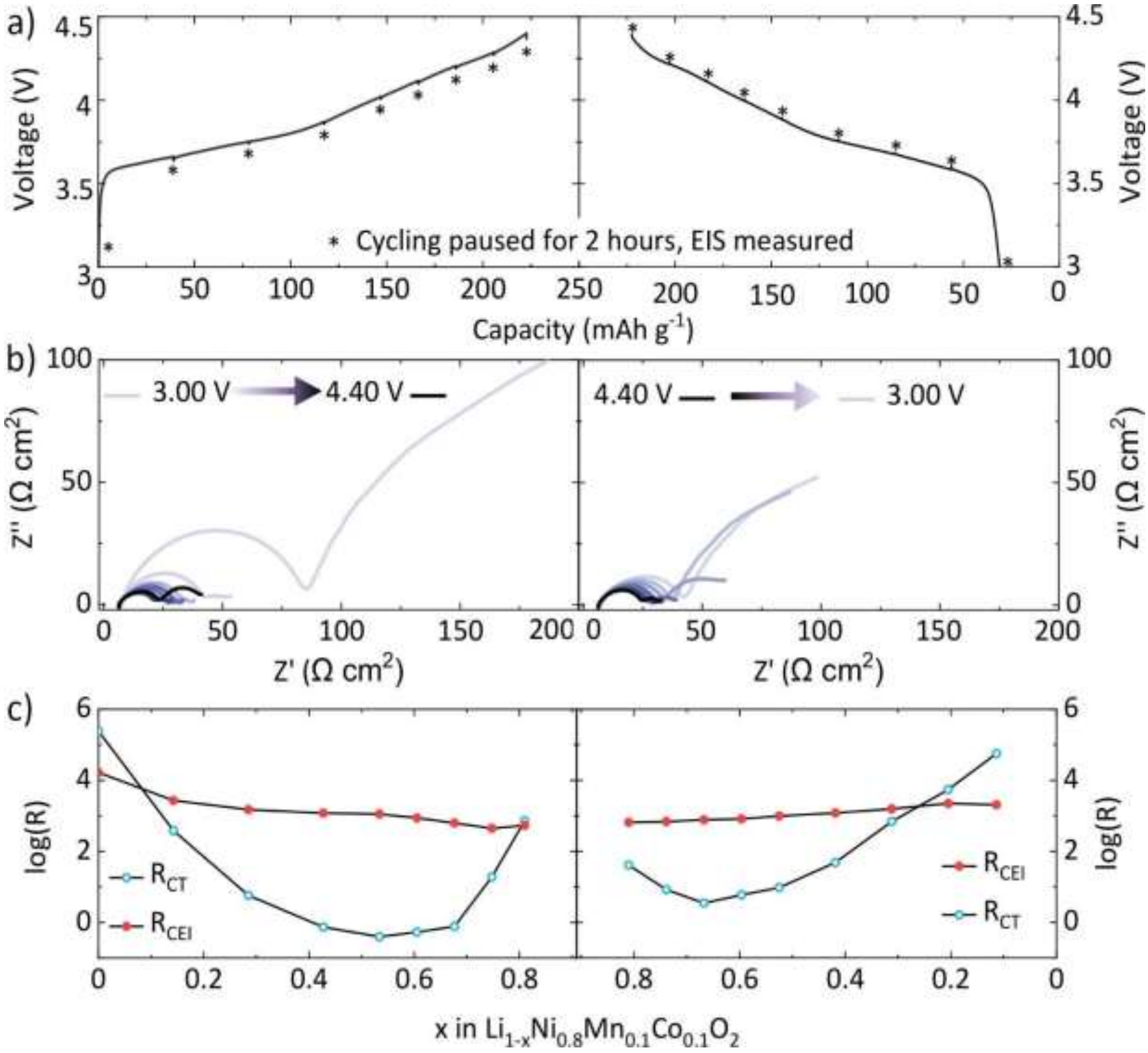

Figure S8. Electrochemical Impedance Spectroscopy (EIS) measurement of Li/NMC811 within a Swagelok cell. (a) The charge/discharge cycle (C/20), with asterisks marking the points where EIS measurements were taken. (b) Nyquist plots during charge and discharge. (c) The change in resistance of the charge transfer (R_{CT}) and cathode-electrolyte interface (R_{CEI}) components as obtained by equivalent circuit fitting (Figure S9).

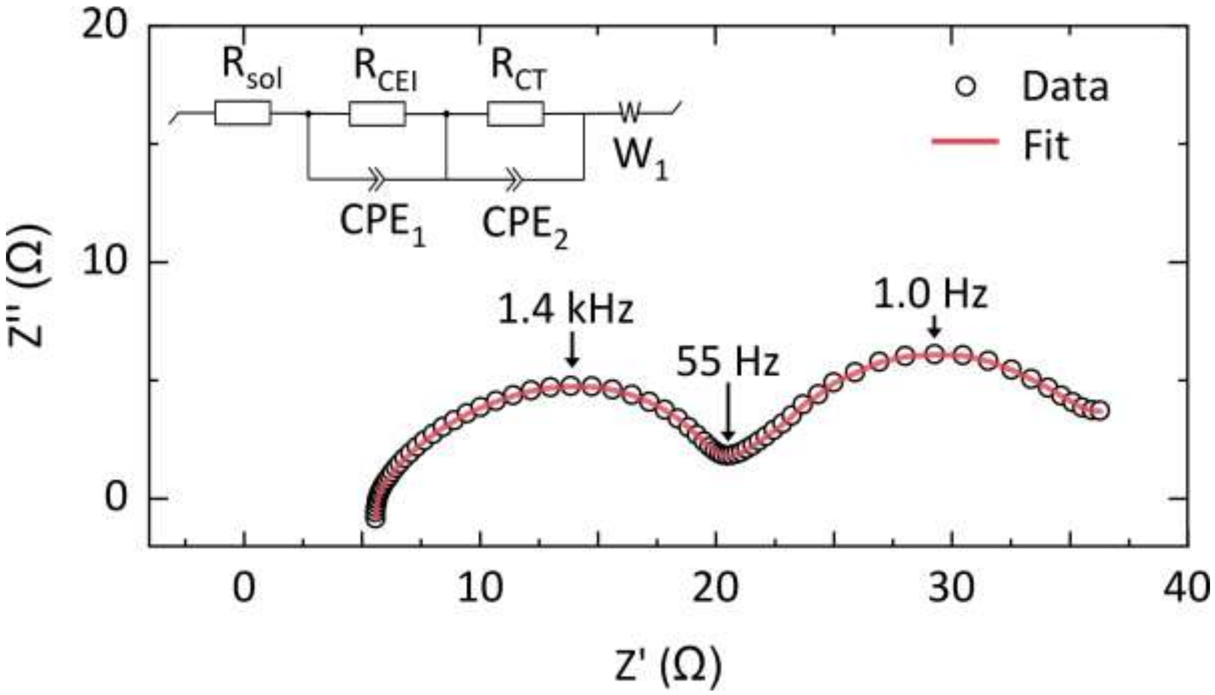

Figure S9. Example of the fitted impedance spectra at 4.4 V for the data shown in Figure S8. An [R_{sol} + R_{CEI}/CPE_1 + R_{CT}/CPE_2 + W_1] equivalent circuit was used, as is common for impedance fitting of NMC half cells.[3] R_{sol} is defined as the solution resistance (between 0 Ω and first semi-circle), R_{CEI} as the cathode/electrolyte interface resistance, and R_{CT} as the charge transfer resistance through NMC secondary particles.

Table S4. Fitted values of resistance and capacitance for the equivalent circuit displayed in Figure S9 across the first cycle of an Li/NMC811 half-cell. These data are presented graphically in Figure 6d. The smaller capacitance of *C*1 (~10^{-6} F) suggests this arises from phenomena at the sample-electrode interface or a passivation layer on the particle surfaces, known as the cathode electrolyte interface (CEI).[4]

Voltage	R_{sol}	R_{CEI}	R_{CT}	Capacitance 1 (μF)	Capacitance 2 (mF)
1st Charge					
OCV (before charge)	6.557	68.03	217.6	1.92	7.62
3.64	6.069	30.94	13.27	2.70	19.2
3.74	5.979	23.91	2.143	3.41	7.68
3.86	5.917	21.71	0.878	3.88	16.7
4.01	5.868	21.13	0.669	4.12	25.1
4.10	5.669	18.94	0.762	4.40	19.1
4.19	5.589	16.45	0.8949	6.23	0.0122
4.27	5.52	14.12	3.591	4.46	7.24
4.38	5.604	17.49	17.49	3.81	9.54
1st Discharge					
4.40	5.566	16.72	5.019	23.6	5.38
4.23	5.604	17.06	2.519	2.73	7.72
4.12	5.64	17.95	1.721	81.8	3.11
4.05	5.671	18.43	2.172	32.7	5.69
3.89	5.717	19.96	2.666	24.2	6.70
3.76	5.74	21.76	5.41	3.63	8.51
3.69	5.799	24.48	17.15	17.7	11.6
3.61	5.84	28.52	42.06	6.27	24.8
3.0	5.826	27.44	116.1	9.16	14.1

Galvanostatic Intermittent Titration Technique (GITT)

The chemical diffusion coefficient, Ds, was evaluated from GITT data by deriving a surface area independent term from the simplified Weppner-Hubbins experesion defined as:[5]

$$S^2 D_s = \frac{4}{\pi\tau} (n_m \, v_m)^2 \, (\frac{\Delta E_s}{\Delta E_t})^2 \qquad \text{Eq. S1}$$

In Eq. S1, S is the active material surface area, τ is the time of the transient current pulse, n_m is the number of moles of the active material, and v_m is the molar volume of the active material. These are assumed to be constant throughout the experiment. ΔEt and ΔEs are defined above as originating from the GITT measurement itself. ΔEt is the difference in voltage during the current pulse period, while ignoring the IR drop component (vertical rise in voltage). ΔEs is the difference in voltage at the end of consecutive relaxation steps. For this method to be valid, we ensure that $\tau << R^2/D_s$, where R is the diffusion length, which is in this case considered to be approximately equal to the average radius of a the secondary particles.

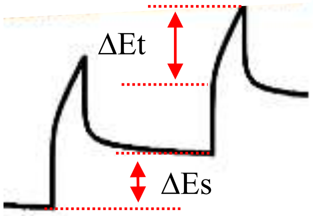

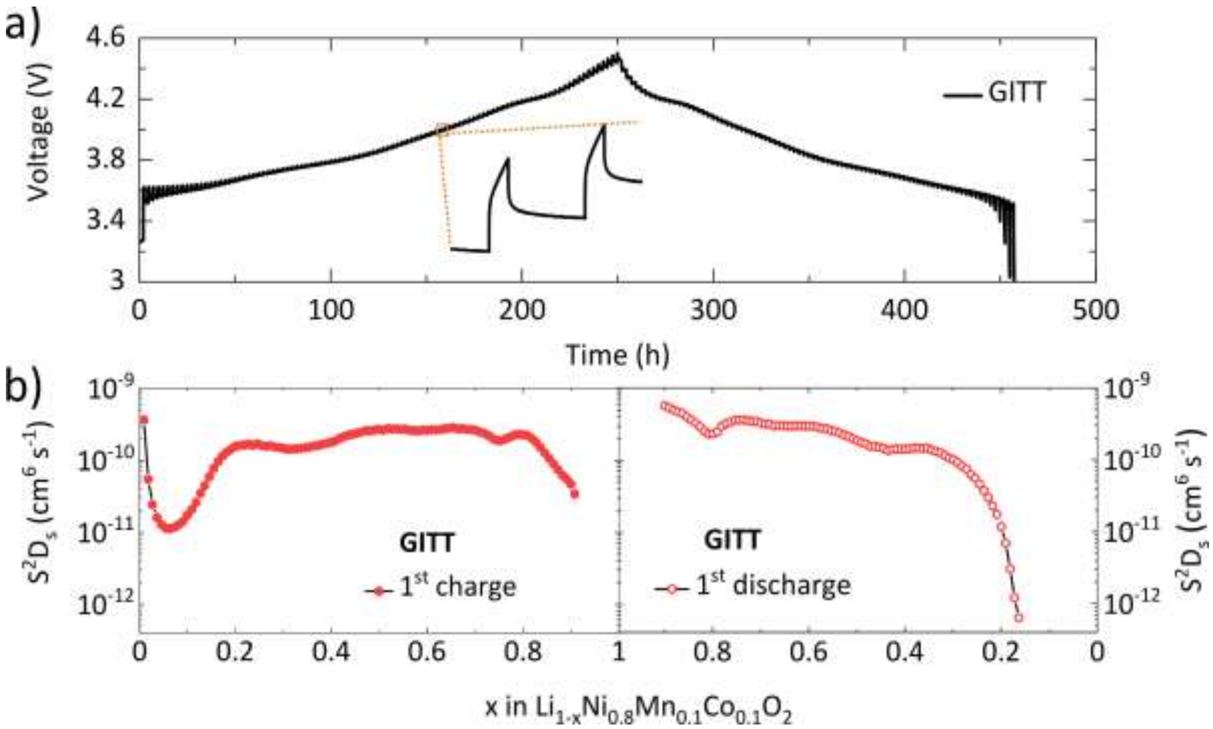

Figure S10. (a) Galvanostatic intermittent titration technique (GITT) measurement of a NMC811 | Li cell, using a 30-minute current pulse and two-hour relaxation period. (b) The surface independent chemical diffusion coefficient (S^2D_s) across the charge and discharge cycle. This data is shown again for comparative purposes in Figure 6e.

Muon fitting parameters overlayed for charge and discharge

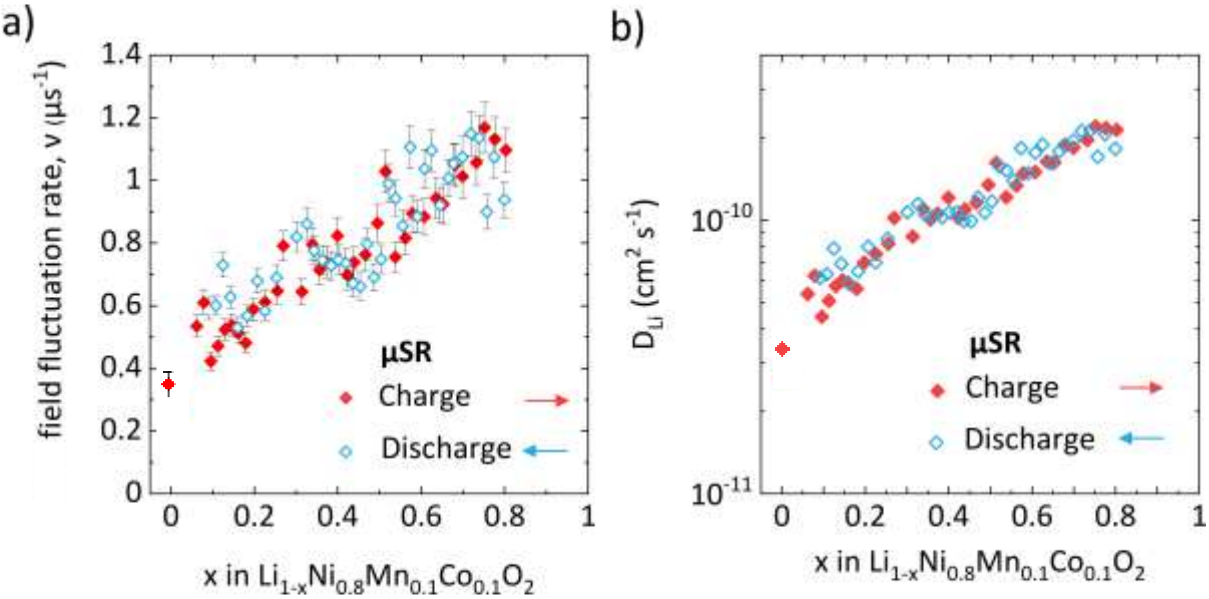

Figure S11. Overlay of the charge and discharge values of (a) the field fluctuation rate and (b) the Li^+ diffusion coefficient for the first cycle of NMC811, obtained via μSR. The charge can be read from left to right and the discharge from right to left. The overlay displays little difference between either parameter between charge and discharge, although the discharge does not reach low *x* values because of the irreversible capacity loss.

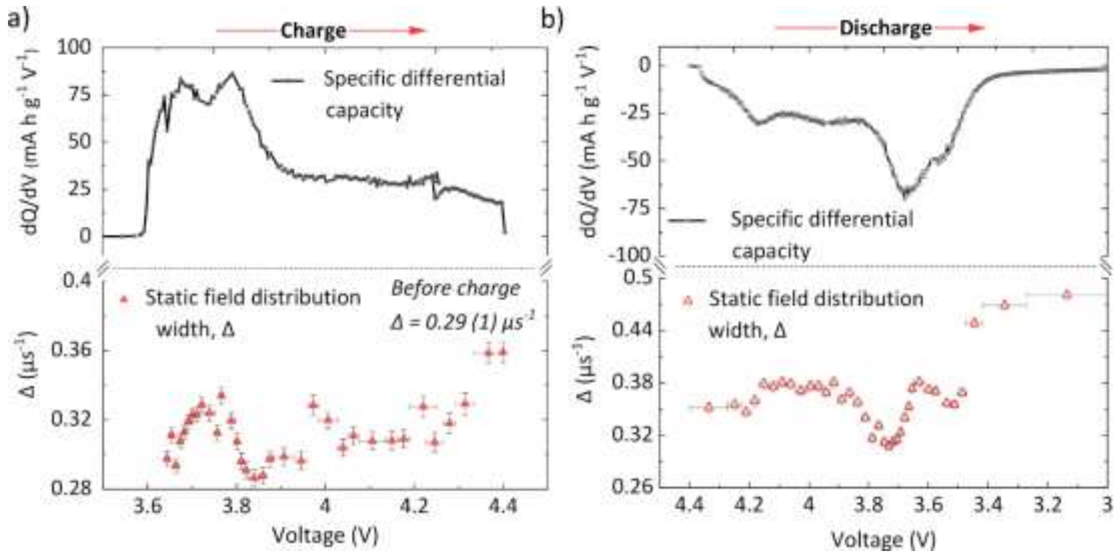

Figure S12. (a) The static field distribution data seen in Figure 7, showing the charge (top) and discharge data (bottom). (b) Full dQ/dV plot for the first cycle of the cell used at the μSR beamtime (also given in Figure 7 on separate plots but replotted here for clarity). (c) Cyclic voltammetry scan of a Li | NMC811 cell recorded with a scan rate of 0.1 mV s^{-1}.

Dipolar Field Calculations and Muon Site Discussion

To better understand the muon stopping site in NMC811, dipolar field calculations were performed to compare theoretical with experimental Δ values. These were completed in a similar manner to the method previously used by our group,[6] using Eq. S2.[7] The unit cell size of z ≤ 0.5 from our previous study was increased to z ≤ 1.0 for these calculations.

$$\Delta^2 = \frac{2}{3}\mu_0^2\gamma_\mu^2\hbar^2\sum_i \frac{I_i(I_i+1)\gamma_i^2}{r_i^6} \qquad \text{(Eq. S2)}$$

In Eq. S2, γ_μ is the muon's gyromagnetic ratio, I_i and γ_i are the spin and the nuclear gyromagnetic ratio of the ith nucleus, respectively, and r_i is the distance between the ith nucleus and the muon site. This was calculated over the full unit cell for NMC811. The muon is predicted as roughly 1 Å away from any oxygen atom in the structure.

Table S5. The isotopes of the elements present in NMC811 which will have an influence on Δ.[8] Isotopes with no significant nuclear moment are not shown. Li is predicted to have a large effect on Δ, with Ni not so much. Although both Mn and Co have isotopes with large nuclear moments and 100% abundance, these are only 0.1 per formula unit and hence have limited influence. O will have very little influence due to the low abundance of any isotope with a non-zero moment. However, the influence of any element is dependent on the specific stopping site of the muon.

**$LiNi_{0.8}Mn_{0.1}Co_{0.1}O_2$**			
**Isotope**	**Nuclear spin (*I*)**	**Magnetic moment (μ/μ_N)**	**Relative abundance**
^{6}Li	1	0.822	7.6 %
^{7}Li	3/2	3.26	92.4 %
^{59}Co	7/2	4.627	100 %
^{61}Ni	3/2	−0.75	1.1 %
^{55}Mn	5/2	3.45	100 %
^{17}O	5.2	−1.89	0.04 %

To enable firm comparisons between calculated and experimental Δ values, an understanding of the muon site is imperative. Δ calculations were thus performed as function of lithium content (i.e., during cycling), where the Li content and the lattice parameters from the *operando* XRD experiment (Figures S6, S6) were used. A range of ≥150 sites across the unit cell which had a Δ value between 0.29 - 0.31 μs^{-1} at OCV were chosen as possible sites due to their similarity to the experimental value of 0.3 ± 0.01 μs^{-1} before cycling. Potential muon stopping sites were chosen as ~1 Å from an O atom. The Δ values for the same sites were then calculated at different lithium contents to compare with experimental values.

Figure S13b shows the Δ value at these selected sites using the lattice parameters and Li content at $x = 0.6$ (~4.1 V). Near the Li sites in the structure, Δ is at a low value in comparison to the OCV value of ~0.3 μs^{-1} before cycling, while in contrast, the sites close to transition metal ions remain much closer to the experimental value of Δ, strongly indicating that muons prefer to reside within the TM layers.

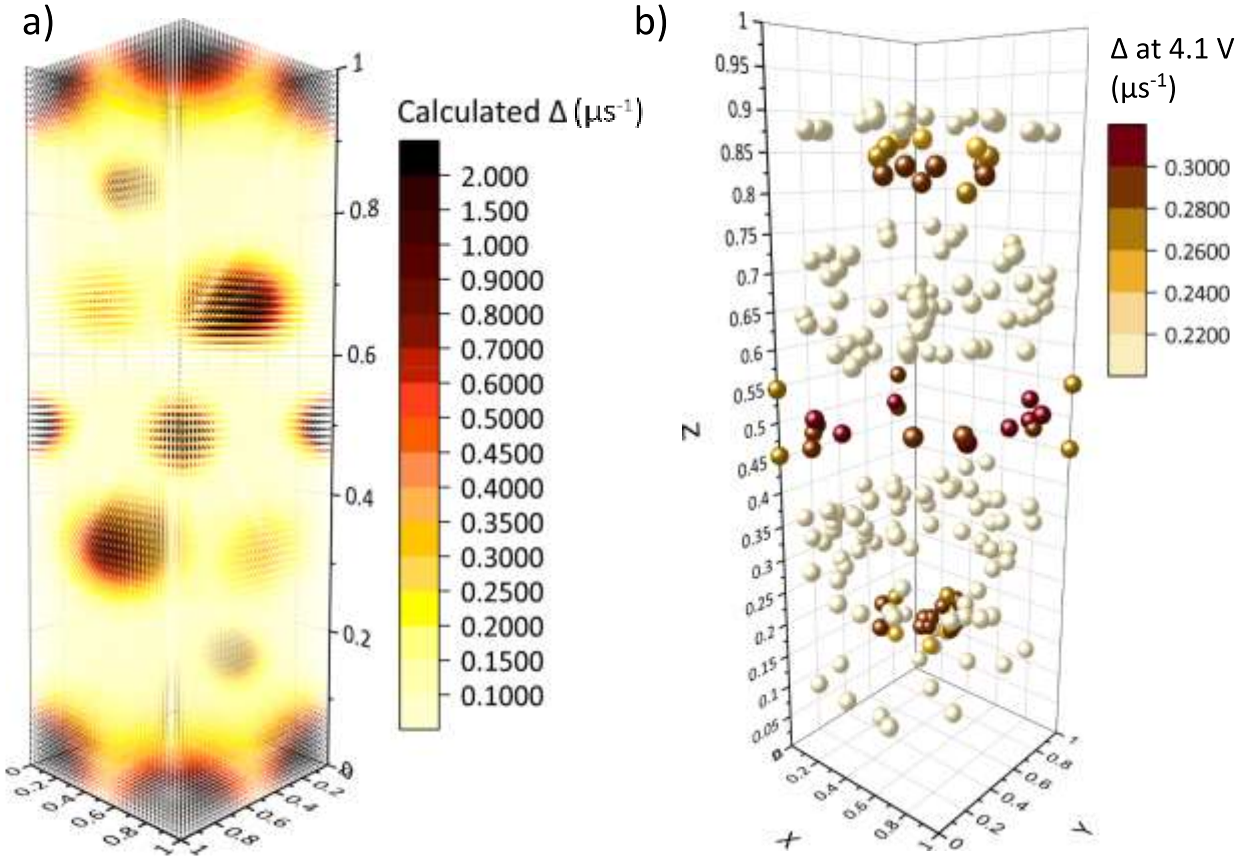

Figure S13. (a) Visual depiction of Δ across the unit cell of NMC811 at full Li occupancy. The Li layer is at $z = 0$ and $z = 1$, while the TM layer is at $z = 0.5$. As Δ^2 is inversely proportional to r^6, near any atomic sites Δ scales rapidly to values >> 2 (shown in black). To match with experimental values, predicted muon sites are within the orange regions. (b) Δ values at 4.1 V (0.4 Li p/f.u.) for muon sites chosen as matching the experimental Δ at OCV (full Li occupancy). It is obvious that for most sites, Δ is far lower than what would be expected from experiment (~0.31 μs^{-1} at 4.1 V). This is perhaps expected due to the lower Li content. Only near the transition metal ions does Δ reflect the experimental observations. This indicates that the muon stopping site is likely within the TM layer and is not heavily influenced by Li content. This agrees well with DFT predictions of the muon site in other layered materials.[9]

Table S6. Calculated Δ values during charging as partially shown in Figure S13b. Sites at OCV which were ~1 Å from an O atom and had a Δ value of between 0.29 - 0.31 μs^{-1}, matching experimentally obtained values. Δ retains its value during charging much better for sites in the TM layer. Δ is not predicted to be too heavily influenced by the contraction of the *c*-axis at high voltage. Selected sites are shown for brevity.

**Layer**	***x***	***y***	***z***	**Δ at OCV**	**Δ at 4.1 V**	**Δ at 4.4 V**
Li	0.600	0.725	0.113	0.294	0.186	0.144
Li	0.150	0.225	0.124	0.297	0.183	0.143
Li	0.200	0.600	0.134	0.295	0.247	0.249
TM	0.450	0.875	0.155	0.294	0.275	0.271
TM	0.225	0.450	0.165	0.295	0.284	0.279
TM	0.525	0.675	0.165	0.295	0.284	0.279
TM	0.450	0.575	0.175	0.297	0.289	0.287
TM	0.425	0.875	0.175	0.297	0.289	0.287
TM	0.150	0.550	0.186	0.292	0.281	0.286
TM	0.425	0.575	0.186	0.299	0.288	0.293
TM	0.450	0.600	0.186	0.292	0.281	0.285
TM	0.425	0.850	0.186	0.299	0.288	0.293
TM	0.200	0.600	0.196	0.294	0.275	0.290
Li	0.800	0.325	0.247	0.295	0.179	0.137
Li	0.750	0.600	0.258	0.304	0.188	0.145
Li	0.800	0.775	0.289	0.299	0.194	0.142
Li	0.675	0.275	0.423	0.290	0.176	0.139
TM	1.000	1.000	0.454	0.294	0.271	0.302
TM	0.200	0.025	0.474	0.297	0.296	0.306
TM	0.775	0.975	0.485	0.299	0.305	0.309
TM	0.225	0.025	0.515	0.299	0.305	0.309
TM	0.975	0.200	0.515	0.299	0.305	0.309
TM	0.775	0.975	0.515	0.299	0.305	0.309
TM	0.825	0.025	0.526	0.297	0.296	0.306
TM	0.975	0.175	0.526	0.297	0.296	0.306
TM	0.025	0.825	0.526	0.297	0.296	0.306
TM	0	0	0.546	0.294	0.271	0.302
Li	0.975	0.625	0.608	0.299	0.191	0.143
Li	0.600	0.400	0.660	0.291	0.194	0.138

Li	0.900	0.250	0.701	0.296	0.195	0.141
Li	0.225	0.525	0.753	0.300	0.182	0.143
TM	0.800	0.400	0.804	0.294	0.275	0.290
TM	0.550	0.400	0.814	0.292	0.281	0.286
TM	0.575	0.125	0.825	0.297	0.289	0.287
TM	0.875	0.425	0.825	0.297	0.289	0.287
TM	0.675	0.150	0.835	0.295	0.284	0.279
TM	0.775	0.225	0.835	0.295	0.284	0.279
TM	0.775	0.550	0.835	0.295	0.284	0.279
TM	0.550	0.125	0.845	0.294	0.275	0.271
TM	0.875	0.450	0.845	0.294	0.275	0.271

Supporting Information References

1 K. Märker, P. J. Reeves, C. Xu, K. J. Griffith, and C. P. Grey. *Chem. Mater.*, 31:2545–2554, 2019.

2 J. Li, L. E. Downie, L. Ma, W. Qiu and J. R. Dahn. *J. Electrochem. Soc.*, 162:A1401−A1408, 2015.

3 T. Li and X. Z. Yuan and L. Zhang and D. Song and K. Shi and C. Bock. *Electrochem. Energy Rev.,* 3:43-80, 2020.

4 J. T. S. Irvine, D. C. Sinclair, and A. R. West. *Adv. Mater.*, 2:132–138, 1990.

5 W. Weppner and R. A. Huggins. *J. Electrochem. Soc*., 124, 1569, 1977.

6 I. McClelland, S. G. Booth, H. El-Shinawi, B. I. J. Johnston, J. Clough, W. Guo, E. J. Cussen, P. J. Baker, and S. A. Corr. *ACS Appl. Energy Mater.*, 4:1527-1536, 2021.

7 R. S. Hayano, Y. J. Uemura, J. Imazato, N. Nishida, T. Yamazaki, R. Kubo. *Phys. Rev. B*, 20:850– 859, 1979.

8 N. J. Stone. *At. Data Nucl. Data Tables*, 90:75– 176, 2005.

9 O. K. Forslund, H. Ohta, K. Kamazawa, S. L. Stubbs, O. Ofer, M. Månsson, C. Michioka, K. Yoshimura, B. Hitti, D. Arseneau, G. D. Morris, E. J. Ansaldo, J. H. Brewer, and J. Sugiyama. *Phys. Rev. B*, 102:184412, 2020.
